# Gastrin-Releasing Peptide Receptors Stimulate MAPK-Mediated Growth of Lung Cancer Cells by Transactivating HER4 in a Neuregulin-1, MAP Kinase-Dependent Manner Requiring Activation of the ROS-System

**DOI:** 10.3390/biology14091225

**Published:** 2025-09-09

**Authors:** Terry W. Moody, Irene Ramos-Alvarez, Tatiana Iordanskaia, Samuel A. Mantey, Robert T. Jensen

**Affiliations:** 1 Center for Cancer Training, National Cancer Institute, National Institutes of Health, Bethesda, MD 20892, USA; moodyt@mail.nih.gov; 2 Digestive Disease Branch, National Institutes of Diabetes and Digestive and Kidney Diseases, National Institutes of Health, Bethesda, MD 20892, USA; irene.ramosalvarez@nih.gov (I.R.-A.); tatiana.iordanskaia@nih.gov (T.I.); samuelm@bdg10.niddk.nih.gov (S.A.M.)

**Keywords:** bombesin, GRPR, HER4, lung cancer, neuregulin, proliferation, receptor transactivation

## Abstract

The bombesin (Bn) receptor family [Gastrin-releasing peptide (GRPR/BB2R) and Neuromedin B receptors (NMBR/BB1R)] are G-protein coupled receptors (GPCR’s) with potent growth effects on normal tissues/numerous cancers, often by transactivating the ErbB receptor-tyrosine kinase (RTK) family. Whereas GRPR stimulation transactivates ErbB RTKs EGFR, HER2, and HER3 in non-small cell lung-cancer (NSCLC) cells, its effects on HER4 are unknown. This study was designed to address this question. Of 12 NSCLC’s studied, 75% had HER4 mRNA expression and Western-Blotting. NCI-H522 and NCI-H661-cells had high levels of GRPR, HER4, and the HER4-ligand neuregulin (NRG1). Adding GRP to NCI-H522/NCI-H661-cells activated HER4, shown by its increased phosphorylation (P-HER4). The GRPR antagonists PD176252/BW2258U89 inhibited this increase. In NCI-H661-cells, GRP stimulated the formation of HER4-homodimers and HER2-HER4-heterodimers. Adding GRP to these NSCLC-cells increased P-ERK/P-AKT, which was inhibited by siRNA-HER4, PD176252, and ibrutinib, as well as *N*-acetylcysteine and Tiron, which reduce reactive-oxygen species (ROS). GRP increased secretion of NRG1 from NSCLC-cells, and NRG1 increased P-HER4 and P-ERK, which were impaired by ibrutinib. GRP and NRG1 stimulated proliferation of NSCLC-cells, which was inhibited by PD176252, siRNA-HER4, or ibrutinib and which was mediated by MAPK, not AKT/PI3K, activation. These results show GRPR activation results in HER4 transactivation in a ROS-dependent manner, which stimulates NSCLC-growth through a MAPK-mediated mechanism.

## Simple Summary

The G-protein-coupled receptor (GPCR) bombesin (Bn) receptor family [Gastrin-releasing peptide (GRPR/BB2R) and Neuromedin B receptors (NMBR/BB1R)] has potent growth effects on normal and neoplastic tissues, often by transactivating the ErbB receptor-tyrosine kinase (RTK) family. Whereas GRPR stimulation transactivates ErbB RTKs EGFR, HER2, and HER3 in numerous tissues, its effects on HER4 are unknown. This study was designed to address this question. In 12 non-small cell lung-cancer (NSCLC)-cell line, 75% had HER4 mRNA expression, with NCI-H522 and NCI-H661-cells having the highest levels of GRPR, HER4, and the HER4-ligand neuregulin (NRG1). GRP activated HER4 in these NSCLC-cells and stimulated HER4 homodimer/HER2-HER4 heterodimer formation as well as activating ERK and AKT by a reactive-oxygen species (ROS)-dependent mechanism. GRP stimulated secretion of NRG1 from NSCLC-cells, which activated both ERK/AKT in these cells as well as proliferation of NSCLC-cells, which was mediated by ERK, not AKT/PI3K, activation. These results show GRPR activation results in HER4 transactivation in a ROS-dependent manner, which stimulates NSCLC-growth through a MAPK-mediated mechanism, supporting the conclusion that GRPR and HER4 can have important growth effects on NSCLC cells.

## 1. Introduction

HER4 is a recent member of the ErbB family of receptor tyrosine kinases (RTKs) that has numerous shared features with the other ErbB members. HER4 also has a number of unique features from the other ErbB members (i.e., EGFR/HER1/HER2, HER3) [1,2,3,4]. HER4 interacts with the most cognate ligands of any of the 4 ErbB family members (i.e., 7 total), with an *N*-terminal extracellular ligand binding site for neuregulin (NRG) 1, NRG2, NRG3, and NRG4, as well as binding sites for heparin-binding epidermal growth factor (HB-EGF), epiregulin, and betacellulin, with NRG3 and NRG4 interacting only with HER4 [2,5,6]. HER4 has a single transmembrane (TM) domain followed by a cytosolic domain with TK activity. HER4 is unique relative to EGFR, HER2, and HER3 in that it has 4 splice variants (SVs) that occur at 2 sites: one in the extracellular juxtamembrane region (JM-a, JM-b), and the second alternative splicing site in the cytosolic C-terminus (CYT1 and CYT2) [4,7,8]. The JM-a splice variant of HER4 can be metabolized by tumor necrosis factor α, shedding a HER4 extracellular domain (ECD) [4,9]. The remaining HER4 fragment can be cleaved by gamma secretase into an intracellular domain (ICD) [4,10]. The HER4 ICD domain can translocate into the nucleus, where it alters expression of transcription factors such as signal transducer and activator of transcription (STAT). In various cancers, similar to the other ErbB’s, HER4’s activation can stimulate tumor growth and aggressiveness (osteosarcoma, melanoma, and tumors of the stomach, ovary, colon, thyroid, and CNS [medulloblastomas, ependymoma, and gliomas]) [4,6,11,12,13,14,15,16]. However, unlike the other ERB family members, its activation, in some cancers, can inhibit tumor proliferation/aggressiveness (breast, bladder, pancreatic) [4,6,17,18] depending on how HER4 is metabolized. HER4 function results from a signal input layer (ligands), a signal transformation layer (RTK), and a signal processing layer (intracellular pathway), including through ERK, AKT, JAK, and STAT activation [2,4,6,19,20,21,22].

Similar to other ErbB members, when HER4 is activated, it undergoes a structural shift from a tethered to extended conformation and can form homodimers with itself or heterodimers with any of the other ErbB members [23,24]. The NRG1 gene is large (1.4 megabases), and the type I precursor protein contains a *N*-terminal propeptide (1–19), NRG1 (20–241), and a cytosolic C terminal (242–640). An EGF-like domain is present at (178–222), which binds to HER4 [25]. NRG1 can fuse with other proteins such as CD47 [26]. NRG1 is a 44 kDa glycoprotein, and ADAM17 is important in the shedding of ligands from lung cancer cells [17]. After binding NRG1, HER4 can be phosphorylated at 15 tyrosine residues, including Y1056, which phosphorylates p85 of PI3K, leading to P-AKT, which increases cancer cellular survival [23]. When P-HER4 is phosphorylated, it interacts with SHC, leading to the activation of the RAS, MEK, and ERK pathways, increasing cancer cellular proliferation. HER4 is upregulated in breast, ovarian, and uterine cancer [8,27].

With other members of the ErB RTK family (EGFR, HER2, HER3), activation by phosphorylation can be regulated by numerous G protein-coupled receptors (GPCRs), including those of the Bn family [28,29]. Adding gastrin-releasing peptide (GRP) to head and neck cancer cells increases the tyrosine phosphorylation of the EGFR within minutes in a Src-dependent mechanism [30]. GRP activates Src, which phosphorylates ADAM-17, leading to the shedding of amphiregulin [31]. The GRPR binds GRP and Bn, but not neuromedin B (NMB), with high affinity [32,33]. When activated by an agonist, the GRPR couples to Gq11, causing phosphatidylinositol (PI) turnover, resulting in the production of diacylglycerol, which activates protein kinase (PK) C, and inositol trisphosphate, which causes elevation of cytosolic calcium. Additionally, second messengers altered by Bn-like peptides include: phospholipases (A, D); MAP kinases (MEK, ERK); PI3K/Akt and Src kinases [33]. When lung cancer cells, containing GRPR’s, are antagonized by GRPR antagonists, such as BW2258U89 or PD176252, proliferation is reduced [34,35].

Numerous previous studies have demonstrated that BnR’s are one of the most frequently overexpressed families of G-protein coupled receptors by tumors, such as non-small cell lung cancer (NSCLC) cells [36,37,38,39]. Extensive studies have demonstrated that one of their main mechanisms of stimulation of growth of both cancers and normal tissues is by transactivation of the ErbB RTK family [29,39,40]. BnR regulates transactivation of EGFR/HER1, HER2, and HER3, each of which has been extensively studied [29,39,40,41]. For HER4, which is the newest member of the ErbB family [42], nothing is known about HER4 transactivation by BnRs or other GPCRs, which regulate lung and other cancer growth. Although it is reported that HER4 can be transactivated by various pathophysiological conditions, such as hyperglycemia [43], or by activation of some GPCRs, such as gonadotropin-releasing factor in cultured gonadotroph cells [44], or LPA receptors in mouse blastocysts [45]. For this reason, the aim of the present study was to examine the ability of BnRs to regulate HER4 activation by receptor transactivation and the possible signaling mechanisms involved, as well as its effects on tumor growth. These studies were performed in NSCLC cells because of the prominent role the activation of BnRs has been shown to play in the growth of these cells through the release of endogenous ligands from these cells, playing an autocrine growth role, and the extensive studies showing their complex signaling in the activation of NSCLC tumor growth [33,46].

## 2. Materials and Methods

### 2.1. Materials

Anti-HER2, anti-PY1284-HER4 (P-HER4), anti-HER4, anti-PT202/Y204-ERK (P-ERK), ERK, anti-PS473AKT (P-AKT), anti-GRPR, anti-NRG1, and anti-Tubulin were from Cell Signaling Technology (Beverly, MA, USA). DMEM, RPMI-1640, FBS, Trypsin/EDTA, 4–20% Tris–Glycine gels, geneticin selective antibiotic (G418 Sulfate), and DMSO were from Invitrogen (Carlsbad, CA, USA). West Dura extended substrate and Super Signal West Femto enhanced chemiluminescent detection reagent, Tiron, and Breast Adenocarcinoma (MCF-7) Total RNA were from Thermo-Fisher Scientific (Rock-ford, IL, USA). The stabilized goat anti-rabbit IgG peroxidase conjugate was from Pierce Biotechnology, Inc. (Rockford, IL, USA). Nitrocellulose membranes were from Schleicher and Schuell Bioscience, Inc. (Keene, NH, USA). Sample Buffer Laemlii 2× concentrate was from Sigma-Aldrich (St. Louis, MO, USA). Nonfat milk Ominlok was purchased from AmericanBio (Natick, MA, USA). Pierce™ BCA Protein Assay was from ThermoFisher (Waltham, MA, USA). GRP and BW2258U89 were from Bachem Bioscience Inc. (King of Prussia, PA, USA), and PD176252 was from MedChemExpress (Monmouth Junction, NJ, USA). *N*-acetylcysteine was from MP Biomedicals (Santa Ana, CA, USA). Ibrutinib was from Pharmacyclics, LLC. (Sunnyvale, CA, USA). siRNA HER4 and control-siRNA were from Dharmacon (Lafayette, CO, USA). Human Prostate Total RNA (Prostate Z), Human Brain Total RNA, and Human Brain Cerebellum Total RNA were from ZYAGEN (San Diego, CA, USA).

### 2.2. Methods

#### 2.2.1. Cells Culture

In 12 series of non-small cell lung cancer (NSCLC) cells/tissues [20,47,48,49,50,51,52,53,54,55], which reported protein expression of HER4, the mean ± SEM positivity was 38.8 ± 2.8% [range 0–91%]. Because we wanted to concentrate on HER4-positive NSCLC cell lines, in the present study, we reviewed these studies and others [56,57,58] and selected 12 NSCLC cell lines reported in at least one study to express HER4 protein. NSCLC cell lines NCI-H157, NCI-H322, NCI-H441, NCI-H522, A549, NCI-H661, NCI-H838, NCI-H1155, NCI-H1299, NCI-H2073, Calu-3, and NCI-H2122 (American type tissue collection (ATCC), Gaithersburg, MD, USA) were cultured in DMEM containing 10% fetal bovine serum (FBS) and 1% penicillin/streptomycin at 37 °C in 5% CO_2_/95% air. As a positive control [41,58,59], the cervical cancer cell line C33A was obtained from ATCC. C33A cervical cancer cells were cultured in DMEM with 10% FBS. The cells were split twice weekly 1/10 with trypsin/EDTA. The cells were used in the exponential growth phase and were mycoplasma-free.

#### 2.2.2. RNA Isolation and RT-PCR

Total RNA was isolated from frozen pellets of the human NSCLC cells and the control C33A cell line. Total RNA was prepared using an RNeasy MiniKit (Qiagen, Valencia, CA, USA). DNA samples were treated with DNase Digestion (Qiagen, Valencia, CA, USA). Total RNA (1 μg) was reverse-transcribed using SuperScriptTMIII first-strand synthesis supermix for qRT-PCR (Invitrogen, Waltham, MA, USA), according to the manufacturer’s instructions for complementary DNA (cDNA) synthesis. One microliter of the RT reaction mix containing cDNA was amplified using specific primers for EGFR, HER2, HER3, HER4, NRG1, NRG2, NRG3, NRG4, or HER4 splice variants (SVs) (JM-a, JM-b, CYT1, and CYT2) (Table 1).

The reaction mixture contained the HotStarTAq^®^ Master Mix Kit (Qiagen, Valencia, CA, USA), according to the manufacturer’s instructions. Amplification for all PCR reactions included an initial cycle of 95 °C for 15 min, followed by 35 cycles of denaturation at 94 °C for 30 s, annealing at 60 °C for 30 s, and extension at 72 °C for 1 min. After the final cycle, all PCR reactions concluded with a 10 min extension at 72 °C. The PCR products were analyzed on a 3% agarose gel and visualized by ethidium bromide staining. β-Actin was used as an internal control. The primers used for neuregulin’s (NRGs), RTKs, and HER4 SVs are indicated in Table 1.

#### 2.2.3. Western Blotting and Immunoprecipitation

Cells were placed in 10 cm dishes and placed in SIT medium (RPMI-1640 containing 5 μg/mL bovine insulin, 10 μg/mL apo-transferrin, and 5 × 10^−8^ M Na_2_SeO_3_). Inhibitors such as PD176252 (0.1 and 1 μM), BW2258U89 (0.1 and 1 μM), ibrutinib (0.001 and 0.01 μM), 5 mM Tiron, or 10 mM *N*-acetylcysteine were added for 1 h. Then 0.1 μM GRP, or 10 ng/mL, NRG1 was added for 5 min at 37 °C. The cells were rinsed twice with PBS, and proteins were extracted with lysis buffer (0.5 mL of 50 mM Tris-HCl (pH7.4), 150 mM NaCl, 1% NP-40, 1% sodium deoxycholate, 1 mM PMSF, 0.2 mM sodium vanadate, and 0.5 mM EGTA). The lysate was sonicated and centrifuged at 10,000× *g* for 5 min, and the supernatant protein concentration was determined using the BCA reagent. For immunoprecipitation, 600 μg of protein was incubated with 4 μL of anti-HER2 or anti-HER4 antibody for 30 min, followed by overnight incubation with 20 μL of protein A/G agarose overnight. Samples were washed 3 times in RIPA buffer, resuspended in 12 μL of 2× SDS Laemmli buffer, and boiled before electrophoresis. The blots were probed with anti-P-HER4 antibody, and HER4 homodimers or HER2/HER4 heterodimers were determined.

For other protein determinations, 6 well plates of NCI-H522 or H661 cells were placed in SIT medium for 2 h with inhibitors, and then GRP or NRG1 was added. After 5 min, the cells were washed twice in PBS and extracted with 150 μL of Lysate buffer. The lysate was sonicated and centrifuged at 10,000× *g* for 5 min, and the supernatant protein concentration was determined using the BCA assay. Protein (40 μg) was separated on a 4–20% Tris/glycine gel and transferred to nitrocellulose membranes. The membranes were treated with 5% milk in TBS, containing 0.05% Tween-20, and treated with primary antibody (1:1000) overnight at 4 °C, followed by secondary antibody (1:1000) for 1 h at 25 °C. The blots were developed with SuperSignal West Fempto chemiluminescent substrate. The intensity of the protein bands was measured using GeneTools software 4.03.05.0 from Syngene (Frederick, MD, USA), which was assessed in the linear detection range. Tubulin was loaded as a control for Western Blot to normalize the levels of protein detected in each blot.

Selected concentrations and incubation times of the different stimulants and inhibitors used in the different assays of the present study were established in preliminary experiments.

Original blots used in this study are summarized in Supplementary Figure S1.

#### 2.2.4. Transfection with siRNA

NCI-H522 or NCI-H661 cells (10^5^) were placed in 6 well plates. When the cells were 50% confluent, they were transfected with 10 nM siRNA HER4, or control-siRNA, in Opti-MEM medium, using Lipofectamine (Invitrogen, Carlsbad, CA, USA). After 48 h, the cells were serum starved for 24 h before conducting experiments.

#### 2.2.5. NRG1-β1 ELISA

NRG1-β1 was detected by ELISA (RayBiotech; Norcross, GA, USA). Cells in 96-well plates were treated with GRP in the presence or absence of PD176252. Diluted conditioned media, from NCI-H522 or H662 cells, was added to 96-well plates and washed 4 times after a 2.5 h incubation. Biotinylated antibody was added for 1 h and then washed 4 times. Streptavidin solution was added for 45 min and washed 4 times. TMB one-step substrate reagent was added, and after 30 min stop solution was added to each well. The samples absorbance was determined in each well at 450 nm.

#### 2.2.6. Proliferation Assays

The growth of NSCLC cells was determined using the MTT and clonogenic assays. For the MTT assay, NCI-H522 or NCI-H661 cells (0.5 × 10^5^) were placed in 96-well plates, with 100 μL of SIT medium, in the presence or absence of PD176252 or ibrutinib. After 24–48 h, 15 μL of MTT (1 mg/mL) was added, and 150 μL of DMSO was added after 4 h. The absorbance was determined at 570 nm. In the clonogenic assay, the base layer contained 3 mL of 5% FBS in SIT medium with 0.5% agarose in 6-well plates. The top layer contained 3 mL of SIT medium with 0.3% agarose (FMC, Rockford, ME, USA), 0.5 × 10^5^ NCI-H522 cells, and GRP (0.1 μM), PD176252 (1 μM), or ibrutinib (0.01 μM), and siRNA HER4. Triplicate wells were plated, and after 2 weeks, 1 mL of p-iodonitrozolium violet was added, and after 16 h, the number of colonies larger than 50 μm in diameter was counted using an Omnicon image analysis system.

#### 2.2.7. Statistics

All experiments were performed at least three times. Data are presented as means ± S.D. or means ± S.E. and were analyzed with single-factor ANOVA analysis (to study differences between the means of two or more independent groups), using Excel descriptive statistics. *p* values < 0.05 were considered significant.

## 3. Results

### 3.1. RT-PCR of Cell Line Extracts

**Figure 1:**
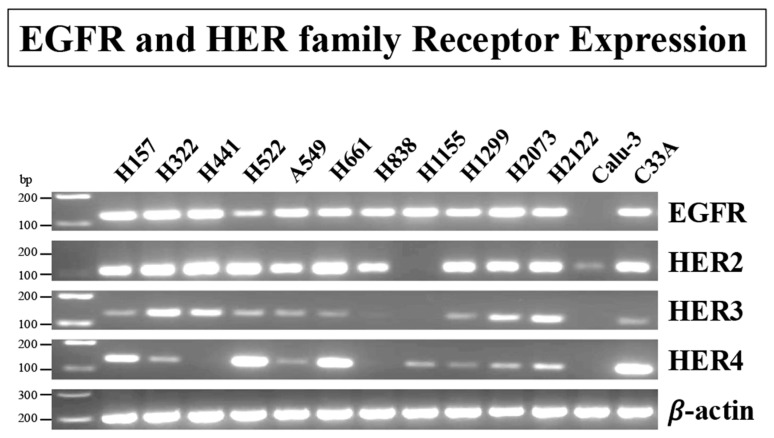
Expression of the EGFR/HER-receptor family in human lung cancer and a cervix cancer cell-line. RT-PCR was performed with 12 non-small cell lung cancer (NSCLC) cell lines and one cervical cancer cell line (C33A) to evaluate the expression of EGFR/HER2, HER3, and HER4-receptor mRNA. β-Actin was used as a loading control. Primers used are shown in Table 1, and experimental conditions are as described in Materials and Methods. This experiment is representative of 2 others. The original western blot images can be found in Supplementary Figure S1.

The presence of the ErbB family members was investigated first in non-small cell lung cancer (NSCLC) cell lines by RT-PCR. EGFR mRNA is detected in 11/12 cell lines (Figure 1).

HER2 mRNA is detected in 11/12 cell lines, and HER3 as well as HER4 PCR products are present in 9/12 cell lines (Figure 1). From the 9 cell lines in which HER4 expression was detected, HER4 mRNA is most abundant in H157, NCI-H522, and NCI-H661 cells, which have high levels of EGFR and HER2 but low levels of HER3. As a control, equal amounts of β-actin mRNA are detected in all cell lines. The results indicate that most NSCLC cells have mRNA for EGFR, HER2, HER3, and HER4.

### 3.2. Western Blot of Cell Line Extracts

**Figure 2:**
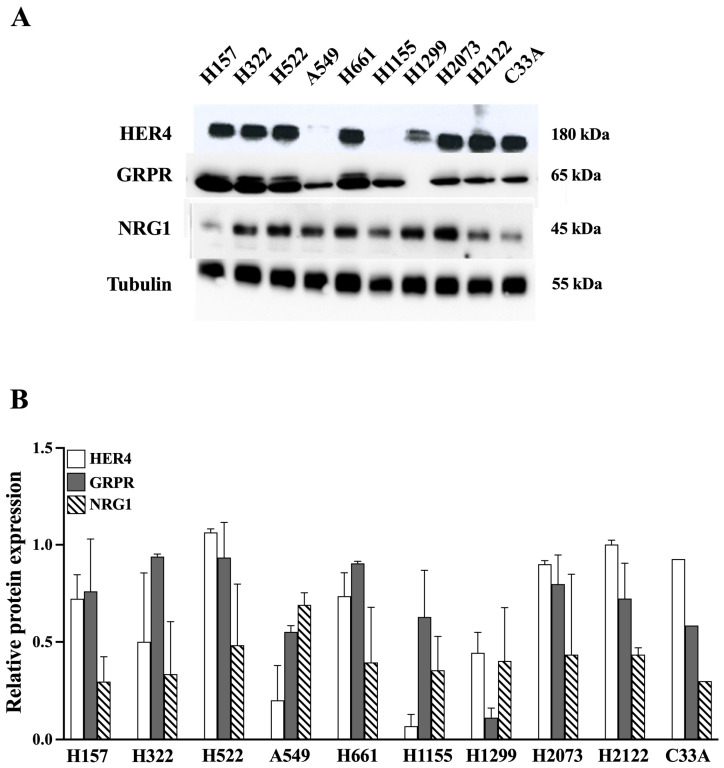
Protein expression of HER4, GRPR, and NRG1 in 9 NSCLC cell lines as well as the cervix cancer cell line C33A. (**A**) Representative blot of HER4, GRPR, NRG1, and Tubulin. (**B**) Relative protein expression of HER4, GRPR, and NRG1. The cell lysates were subjected to Western blotting and analyzed using anti-HER4, anti-GRPR, anti-NRG1, and, as a loading control, anti-tubulin. Bands were visualized using chemiluminescence. Results are expressed as the ratio of the protein expression compared to tubulin. This experiment is representative of 2 others. Abbreviations: GRPR, Gastrin-Releasing Peptide Receptor; NRG1, Neuregulin-1. The original western blot images can be found in Supplementary Figure S2.

Nine NSCLC cell lines were analyzed for HER4, GRPR, NRG1, and tubulin protein, as well as one control, C33A cervical cancer cells (Figure 2)

The highest HER4 protein levels (180 kDa) are present in cell lines NCI-H157, NCI-H322, NCI-H522, NCI-H661, NCI-H2073, NCI-H2122, and in C33A cervical cancer. Moderate levels of NRG1 protein (45 kDa) were detected in NCI-H322, NCI-H522, A549, NCI-H661, NCI-H1299, NCI-H2073, and NCI-H2122. High levels of GRPR (65 kDa) were present in NCI-H157, NCI-H322, NCI-H522, and NCI-H661, with moderate levels in A549, NCI-H1155, NCI-H2073, and NCI-H2122 cells. Equal amounts of the tubulin control are present in each cell line. Because NCI-H522 and NCI-H661 cells have HER4, NRG1, and GRPR, they were used to study HER4 transactivation.

### 3.3. GRP and HER4 Phosphorylation

**Figure 3:**
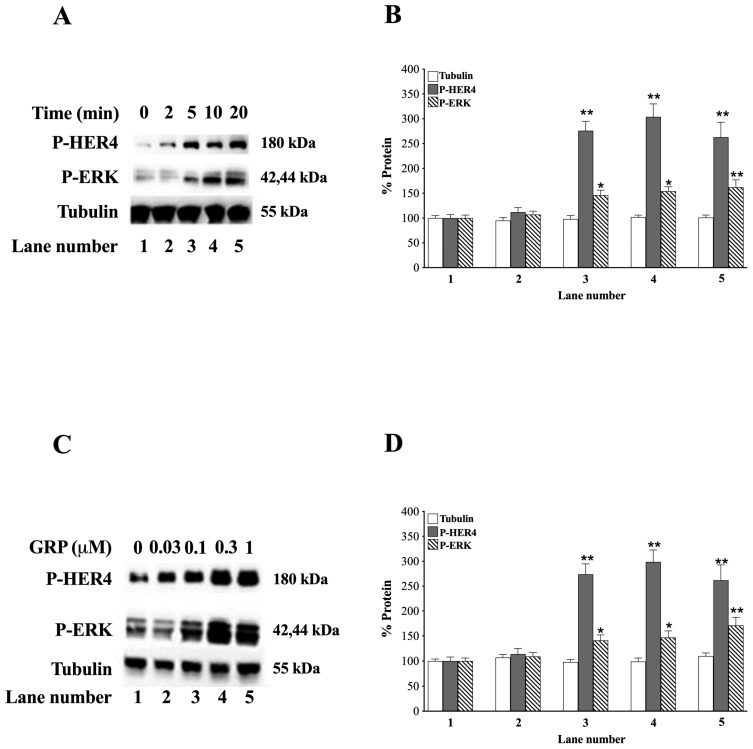
Time course and dose-response of GRP for stimulating activation of HER4 and ERK in NCI-H661 cells. (**A**) The effects of GRP (0.1 μM) were investigated at 0, 2, 5, 10, and 20 min on P-HER4, P-ERK, and tubulin using NCI-H661 cells. (**B**) The % increase caused as a function of time is indicated for tubulin, P-HER4, and P-ERK. (**C**) The effects of GRP were investigated as a function of dose. (**D**) After 10 min 0, 0.03, 0.1, 0.3, and 1 μM GRP were added to NCI-H661 cells, and the % increase as a function of dose is indicated for tubulin, P-HER4, and P-ERK. The mean value ± S.E. of 3 determinations is indicated; *, *p* < 0.05 vs. 0 min or 0 μM GRP; **, *p* < 0.01 vs. 0 min or 0 μM GRP. The exact *p*-values are shown in the text in 3.3, the GRP and HER4 phosphorylation section of the paper. Abbreviations: GRP, Gastrin-Releasing Peptide; min, minute; P-HER4, PY1284-HER4; P-ERK, PT202/Y204-ERK. The original western blot images can be found in Supplementary Figure S3.

The time course and dose of GRP needed to phosphorylate P-HER4 and ERK were investigated. Adding 0.1 μM GRP to NCI-H661 cells increases P-HER4 strongly at 5 min or later (Figure 3A).

After adding GRP to NCI-H661 cells at 5, 10, and 20 min P-HER4 increases significantly to 276, 304, and 263%, respectively (*p* = 0.006, *p* = 0.007, and *p* = 0.009 vs. 0 min, respectively) (Figure 3B). Similarly, GRP increases P-ERK significantly to 146, 154, and 162% after 5, 10, and 20 min, respectively (*p* = 0.015, *p* = 0.02, and *p* = 0.009 vs. 0 min, respectively) (Figure 3A,B). The increase in P-HER4 or P-ERK, caused by GRP, is dose-dependent (Figure 3C). GRP 0.03 μM has little effect; however, GRP doses at 0.1 μM, or greater, strongly increased P-HER4. Adding GRP to NCI-H661 cells increases P-HER4 to 274, 299, and 262% at 0.1, 0.3, and 1 μM doses, respectively (*p* = 0.005, *p* = 0.007, and *p* = 0.005 vs. 0 μM, respectively) (Figure 3D). Similarly, P-ERK increases in a dose-dependent manner from 0.1 μM (*p* = 0.03, *p* = 0.04, and *p* = 0.008 vs. 0 μM, respectively) (Figure 3C,D). As a control, GRP has no effect on total tubulin (Figure 3).

### 3.4. GRP Receptor Antagonists

**Figure 4:**
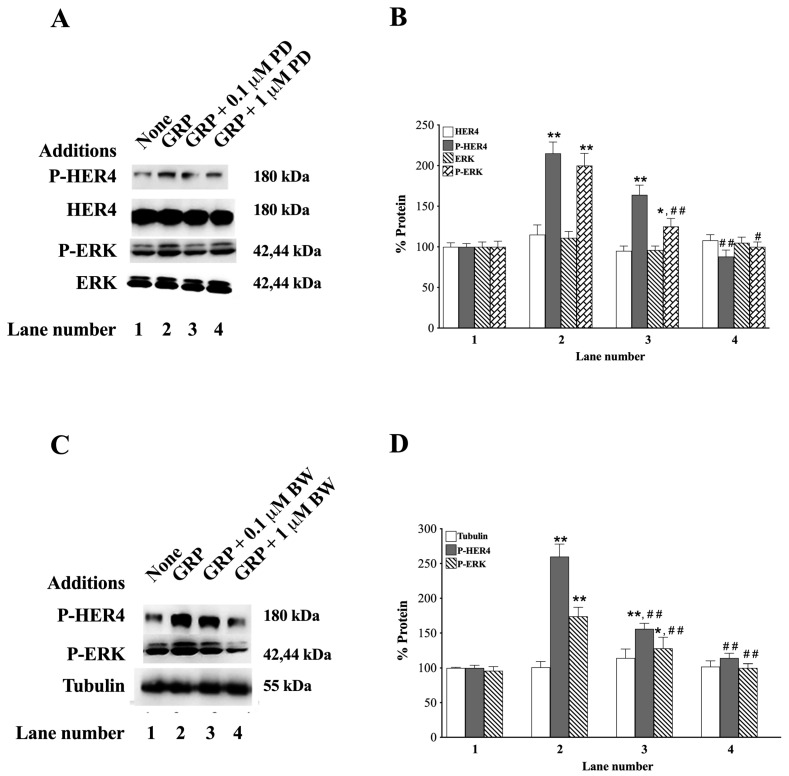
The ability to antagonize the GRPR was investigated using the small molecule receptor antagonist, PD176252, or the specific GRPR receptor peptide antagonist, BW2258U89, in NCI-H661 cells. (**A**) PD176252 has a dose-dependent effect on 0.1 μM GRP stimulation of P-HER4 or P-ERK. (**B**) The % P-HER4, P-ERK, HER4, or ERK altered by GRP is indicated as a function of PD176252 dose. (**C**) BW2258U89 at both 0.1 and 1 μM doses inhibited the 0.1 μM GRP-induced increase in P-HER4 and P-ERK. (**D**) The % tubulin, P-HER4, and P-ERK were determined as a function of BW2258U89 dose. The mean value ± S.E. of 3 determinations is shown; *, *p* < 0.05 vs. None; **, *p* < 0.01 vs. None; #, *p* < 0.05 vs. GRP; ##, *p* < 0.01 vs. GRP. The exact *p*-values are shown in the text in 3.4, the GRP receptor antagonists section of the paper. Abbreviations: BW, BW2258U89; GRP, Gastrin-Releasing Peptide; P-HER4, PY1284-HER4; P-ERK, PT202/Y204-ERK; PD, PD176252. The original western blot images can be found in Supplementary Figure S4.

The ability to antagonize the GRPR was investigated using the small molecule peptoid receptor antagonist, PD176252 [60,61], or the GRPR peptide receptor antagonist, BW2258U89 [33]. Figure 4A shows that 1 μM, but not 0.1 μM, PD176252 impairs the ability of GRP to increase P-HER4 in H661 cells.

Figure 4B shows that adding GRP to NCI-H661 cells increases P-HER4 and P-ERK to 215 and 200%, respectively (*p* = 0.002 and *p* = 0.009 vs. None, respectively), and the P-HER4 increase was significantly inhibited by 1 μM PD176252 (*p* = 0.001 vs. GRP), while the P-ERK increase was significantly inhibited by both concentrations of PD176252 (0.1 μM: *p* = 0.09 vs. GRP; and 1 μM: *p* = 0.001 vs. GRP). As a control, PD176252 or GRP has no effect on total HER4 or ERK in H661 cells (Figure 4A,B). Figure 4C shows that BW2258U89 inhibits P-HER4 and P-ERK phosphorylation. Figure 4D shows that GRP increased P-HER4 and P-ERK to 260% and 174%, respectively (*p* = 0.002 and *p* = 0.009 vs. None, respectively). The increase caused by GRP was totally inhibited by both concentrations of BW2258U89 used (P-HER4: *p* = 0.03 and *p* = 0.008 vs. GRP, respectively. P-ERK: *p* = 0.02 and *p* = 0.002 vs. GRP, respectively).

The antagonists had no effect on the tubulin control (Figure 4C,D). These results show that the GRPR antagonists, PD176252 and BW2258U89, inhibit the ability of GRP to activate GRPR, resulting in decreased stimulation of HER4 transactivation.

### 3.5. siRNA HER4

**Figure 5:**
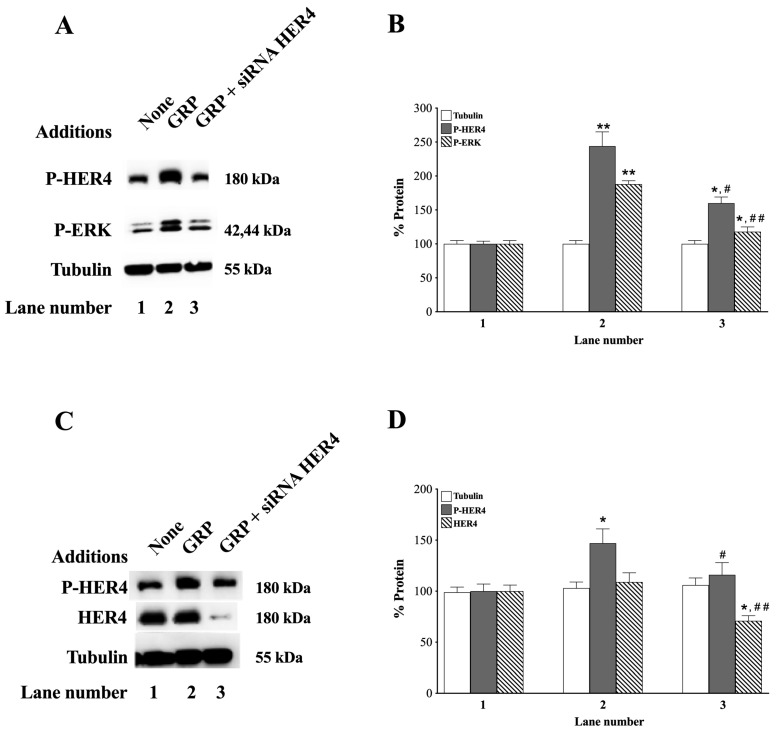
The effect of siRNA HER4 on phosphorylation of HER4 and ERK by GRP in NCI-H661 and NCI-H522 cells. (**A**) siRNA HER4 was added to NCI-H661 for 48 h. NCI-H661cells were placed in SIT medium for 24 h and stimulated with 0.1 μM GRP for 10 min. (**B**) The NCI-H661 cell extracts were assayed for P-HER4, P-ERK, and tubulin. (**C**) siRNA HER4 was added to NCI-H522 cells for 48 h. After treatment with siRNA HER4, NCI-H522 cells were placed in SIT medium for 24 h and stimulated with 0.1 μM GRP for 10 min. T (**D**) The NCI-H522 cell extracts were assayed for P-HER4, HER4, and tubulin. This experiment is representative of 2 others, and the mean value ± S.E is shown; *, *p* < 0.05 vs. None; **, *p* < 0.01 vs. None; #, *p* < 0.05 vs. GRP; ##, *p* < 0.01 vs. GRP. The exact *p*-values are shown in the text in the 3.5. siRNA HER4 section of the paper. Abbreviations: GRP, Gastrin-Releasing Peptide; P-ERK, PT202/Y204-ERK; P-HER4, PY1284-HER4. The original western blot images can be found in Supplementary Figure S5.

The effects of siRNA HER4 were investigated. Adding GRP to NCI-H661 cells increases P-HER4 and P-ERK, which are impaired by siRNA HER4 (*p* = 0.05 and *p* = 0.002 vs. GRP, respectively) (Figure 5A).

GRP increases the P-HER4 and P-ERK to 244 and 188%, respectively (*p* = 0.002 and *p* = 0.006 vs. None, respectively) (Figure 5B). siRNA HER4 had no effect on the tubulin control (Figure 5A,B). Adding GRP to NCI-H522 cells increases *p*-HER4 but has little effect on tubulin (Figure 5C). In NCI-H522 cells, GRP increases *p*-HER4 to 147%, which was decreased by HER4 siRNA (*p* = 0.03 and *p* = 0.14 vs. None, respectively), but had no effect on tubulin (Figure 5D). The results indicate siRNA HER4 impairs the ability of the GRPR activation to regulate HER4 in NSCLC cells (*p* = 0.04 and *p* = 0.008 vs. GRP, respectively).

### 3.6. HER4 Dimerization and the Effect of ROS Inhibitors

**Figure 6:**
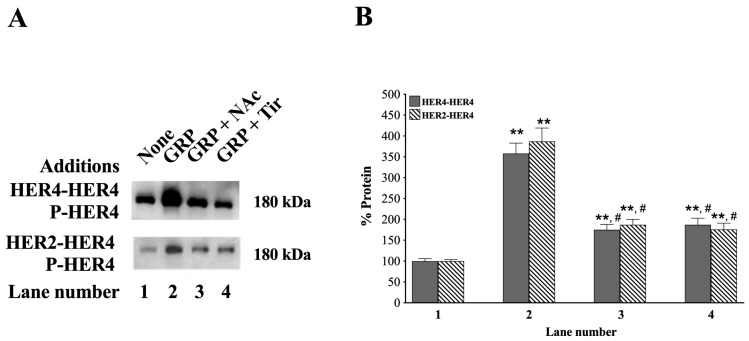
Effect on co-immunoprecipitation of HER2 and HER4 caused by GRP alone, GRP + NAc, and GRP plus Tiron in NCI-H661 cells. (**A**) NCI-H661 cells were treated with *N*-Acetylcysteine (10 mM) or Tiron (5 mM). After 30 min, the cells were stimulated with GRP (0.1 μM) for 10 min and extracted with Lysis buffer. The extracts were immunoprecipitated with either anti-HER4 (top) or anti-HER2 Ab (bottom) overnight in the presence of protein A agarose. The beads were washed with PBS, treated with sample buffer, and the supernatant analyzed by Western blot for P-HER4 presence. (**B**) The % P-HER4 was determined for extracts immunoprecipitated with anti-HER4 and for extracts immunoprecipitated with anti-HER2. The mean value ± S.E. of 3 experiments is indicated; **, *p* < 0.01 vs. None; #, *p* < 0.05 vs. GRP. The exact *p*-values are shown in the text in 3.6. HER4 dimerization and the effect of ROS inhibitors section of the paper. Abbreviations: GRP, Gastrin-Releasing Peptide; NAc, *N*-acetylcysteine; P-HER4, PY1284-HER4; Tir, Tiron. The original western blot images can be found in Supplementary Figure S6.

HER4 can form homodimers with itself or heterodimers with HER2, as well as with the other ErbB family members [1,23,24,62]. Figure 6 (top left) shows that in NCI-H661 cells, after immunoprecipitation with anti-HER4, GRP increased P-HER4, but the increase was impaired by *N*-acetylcysteine (NAc is an antioxidant) [63] or Tiron (superoxide scavenger) [64] (*p* = 0.03 and *p* = 0.03 vs. GRP, respectively).

After immunoprecipitation with anti-HER4, the increase in P-HER4 caused by GRP alone, GRP + NAc, and GRP plus Tiron was 358%, 175%, and 187% (*p* = 0.002, *p* = 0.008, and *p* = 0.006 vs. None, respectively) (Figure 6B). When the NCI-H661 extract was instead first immunoprecipitated with anti-HER2, GRP strongly increased the tyrosine phosphorylation of HER4, which was impaired by NAc or Tir (*p* = 0.01 and *p* = 0.01 vs. GRP, respectively) (Figure 6 bottom left). Treatment of NCI-H661 cells with GRP, GRP with NAc, and GRP plus Tiron increased the % of P-HER4 to 387, 187, and 176%, respectively (*p* = 0.002, *p* = 0.006, and *p* = 0.008 vs. None, respectively) (Figure 6, right). These results indicate that adding GRP to NSCLC cells increases the formation of HER2-HER4 heterodimers and HER4-HER4 homodimers in a ROS-dependent manner.

### 3.7. Effects of Ibrutinib

**Figure 7:**
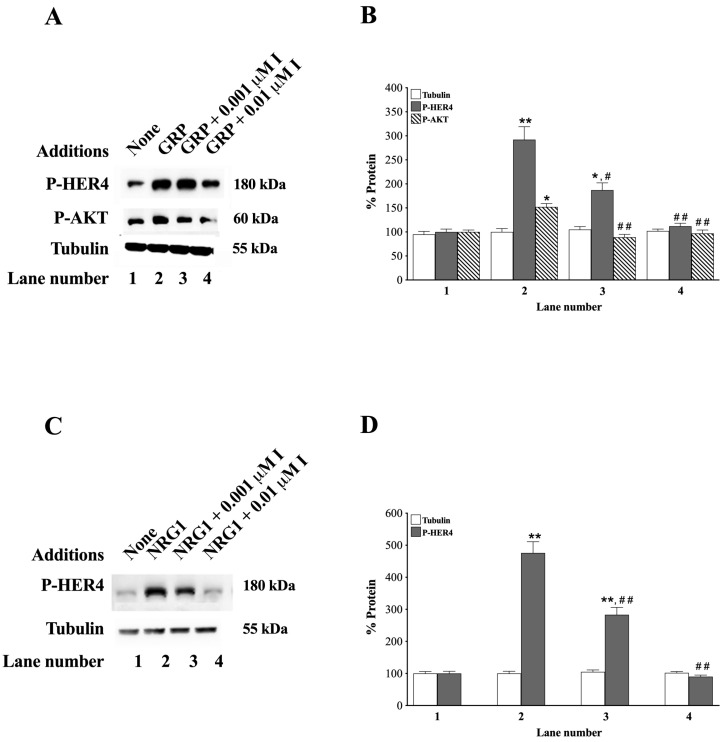
The effects of the tyrosine kinase inhibitor, ibrutinib, on the ability of GRP and NRG1 to increase P-HER4 and P-AKT in NCI-H661 cells. (**A**) The effect of varying doses of ibrutinib to inhibit the effects of GRP on P-HER4, P-AKT, and tubulin was investigated using NCI-H661 cells. (**B**) The % increase in tubulin, P-HER4, and P-AKT was determined in the absence and presence of 0.001 and 0.01 μM ibrutinib, respectively. (**C**) The effect of varying doses of ibrutinib to inhibit transactivation of HER4 caused by adding NRG1 to NCI-H661 cells is indicated. (**D**) The % tubulin and P-HER4 are indicated. The mean value ± S.E. of 3 experiments is shown; *, *p* < 0.05 vs. None; **, *p* < 0.01 vs. None; #, *p* < 0.05 vs. GRP; ##, *p* < 0.01 vs. GRP or NRG1. The exact *p*-values are shown in the text in the 3.7. Effect of Ibrutinib section of the paper. Abbreviations: GRP, Gastrin-Releasing Peptide; I, ibrutinib; P-AKT, PS473AKT; P-HER4, PY1284-HER4. The original western blot images can be found in Supplementary Figure S7.

Ibrutinib (tyrosine kinase inhibitor), which is reported to inhibit HER4 activation as well as a number of other tyrosine kinase receptors, was investigated [6,65,66,67]. It has high affinity for Bruton tyrosine kinase and HER4 (nM range); however, Bruton tyrosine kinase only occurs in low concentrations in NSCLC cells, and any growth effect is inhibitory, not stimulatory [68]. Therefore, we used ibrutinib to investigate the effects of HER4 activation in NCI-H661 cells (Figure 7).

Ibutinib, at 0.001 or 0.01 μM, inhibited GRP-stimulated increases in P-HER4 (Figure 7A). Adding GRP to NCI-H661 cells increases P-HER4 to 292% and P-AKT to 152% (*p* = 0.004 and *p* = 0.012 vs. None, respectively). Ibrutinib, at 0.001 or 0.01 μM, decreases P-HER4 to 187 and 112% (*p* = 0.02 and *p* = 0.001 vs. GRP, respectively), as well as P-AKT to 89 and 95%, respectively (*p* = 0.005 and *p* = 0.007 vs. GRP, respectively) (Figure 7B). Ibrutinib, at a 0.001 and 0.01 μM concentration, impairs the ability of NRG1 to increase P-HER4 (*p* = 0.003 and 0.001 vs. NRG1, respectively) (Figure 7C, Lane 4). Adding NRG1 to NCI-H661 cells increases P-HER4 to 476%, which is reduced by 0.001 or 0.01 μM ibrutinib to 283 and 90%, respectively (*p* = 0.002, *p* = 0.009, and *p* = 0. 12 vs. None, respectively) (Figure 7D). GRP, NRG1, or ibrutinib had no effect on the tubulin control. The results indicate that ibrutinib inhibits the increase in P-HER4 caused by adding GRP or NRG1 to NSCLC cells.

### 3.8. Effects of NRG1

**Figure 8:**
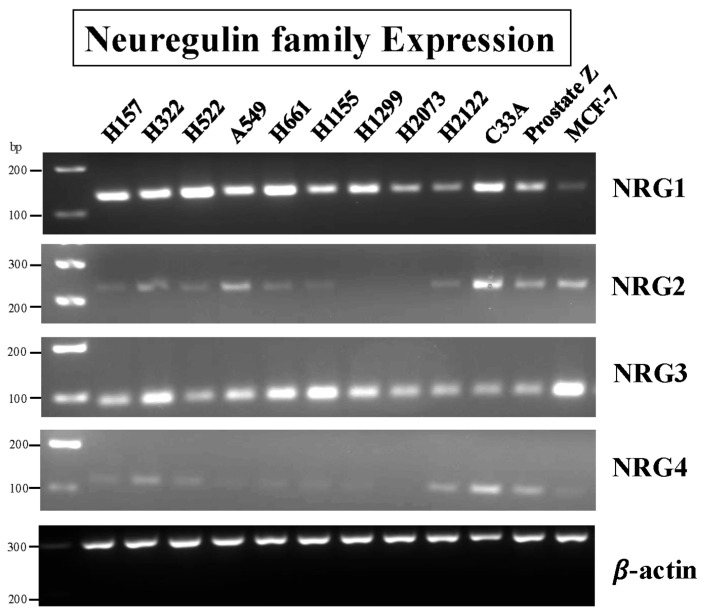
Expression of the Neuregulin family in human NSCLC cell lines, one cervical cancer cell line, one prostate cancer cell line, and one breast cancer cell line. PCR was performed with 9 human lung cancer cell lines, one cervical cancer cell line (C33A), and one prostate (Prostate-Z) and breast cancer cell line (MCF-7) to evaluate the expression of Neuregulin mRNA. β-Actin was used as a loading control. Primers used are shown in Table 1, and experimental conditions are as described in Materials and Methods. This experiment is representative of 2 others. Abbreviation: NRG, Neuregulin. The original western blot images can be found in Supplementary Figure S8.

Neuregulins are proteins that activate HER4 and HER3, but not the EGFR or HER2 [23,25]. Figure 8 shows that NRG1 and NRG3 PCR products are detected in 9/9 NSCLC cell lines.

The NSCLC cell lines NCI-157, NCI-H322, NCI-H522, A549, NCI-H661, H1299, and the control cervical cancer cell line, C33A cells, have high levels of NRG1 mRNA. High levels of NRG3 are detected in the NSCLC cell lines NCI-H522, H661, NCI-H1155, NCI-H1299, and the control MCF7 breast cancer cells. Low levels of NRG2 and NRG4 mRNA are detected in less than one-half of the NSCLC cells. The results indicate that most NSCLC cells have high levels of mRNA for NRG1 (interacts with HER3 and HER4) and NRG3 (interacts only with HER4), but not NRG2 (interacts with HER3) or NRG4 (interacts only with HER4).

The secretion of NRG1β from NSCLC cells was investigated. Table 2 shows that with NCI-H522 cells, GRP (0.1 μM) increases NRG1β secretion, with the supernatant concentration of NRG1β increasing from 140 pg/mL to 235 pg/mL after the addition of GRP. The increase in NRG1β secretion from NCI-H522 cells decreases significantly when GRP plus 1 μM PD176252 is added. Table 2 shows that adding 0.1 μM GRP to NCI-H661 cells also stimulates NRG1β secretion, with the addition of GRP increasing the supernatant concentration of NRG1β from 152 to 322 pg/mL.

The increase in NRG1β secretion from NCI-H661 cells, caused by GRP, is significantly decreased by 1 μM PD176252. The results indicate that the activation of GRPR stimulates secretion of NRG1β secretion from both NCI-H522 and NCI-H661 cells.

### 3.9. Proliferation

**Figure 9:**
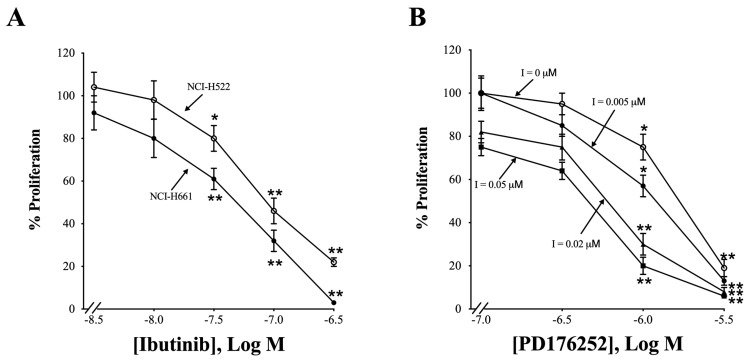
The effects of ibrutinib (TKI) and PD176252 (GRPR antagonist) on NSCLC proliferation. (**A**) The ability of ibrutinib to inhibit the proliferation of NCI-H522 and NCI-H661 cells was investigated as a function of dose. (**B**) The ability of PD176252 to inhibit the proliferation of NCI-H522 cells was investigated as a function of dose in the presence of 0, 0.005, 0.02, and 0.05 μM ibrutinib. The absorbance of 8 samples was determined, and the mean value ± S.E. of 3 experiments is indicated; *, *p* < 0.05 vs. None; **, *p* < 0.01 vs. None. The exact *p*-values are shown in the text in 3.9, the Proliferation section of the paper. Abbreviation: I, ibrutinib.

Figure 9A shows the effects of inhibition of HER4 by the TK inhibitor, ibrutinib, or by the GRPR receptor antagonist, PD176252, on NCI-H522 and NCI-H66 NSCLC proliferation.

With both NSCLC lines, using the MTT assay, ibrutinib has little effect at a 0.003 or 0.01 μM dose but significantly inhibits proliferation at a 0.03, 0.1, and 0.3 μM dose (NCI-H522: *p* = 0.02, *p* = 0.002, and *p* = 0.001 vs. None, respectively. NCI-H661: *p* = 0.006, *p* = 0.001, and *p* = 0.001 vs. None, respectively) (Figure 9A). PD176252 inhibits NCI-H522 proliferation in a dose-dependent manner, with 0.1 or 0.3 μM having little effect (Figure 9B). For NCI-H522 cells, PD176252 inhibits proliferation to 75% and 19%, using a 1 or 3 μM dose, respectively (*p* = 0.02 and *p* = 0.001 vs. None, respectively). Ibrutinib potentiates the effects of PD176252 (Figure 9B). In the presence of 0.005 μM ibrutinib, PD176252 has little effect at 0.1 and 0.3 μM but significantly inhibited proliferation at 1 and 3 μM (*p* = 0.01 and *p* = 0.001, respectively). Ibrutinib had little effect at 0.02 μM but significantly inhibited proliferation at 0.1 and 0.3 μM PD176252 (*p* = 0.008 and *p* = 0.001, respectively). Ibrutinib had little effect at 0.05 μM and PD176252 (0.1 and 0.3 μM), but significantly inhibited proliferation at 0.1 and 0.3 μM PD176252 (*p* = 0.002 and *p* = 0.001, respectively). The results indicate that the ability of PD176252 to decrease NCLC proliferation is increased when ibrutinib is present.

In the clonogenic assay, siRNA HER4 decreases proliferation of NCI-H661 by 67% (Table 3).

PD176252 (10 μM) decreases the growth by 45%, whereas 10 μM ibrutinib reduces the proliferation by 23%. The combination of PD176252 and ibrutinib decreases proliferation significantly by 55%. GRP (0.1 μM) and NRG1 (10 ng/mL) increase colony number to 200% and 222%, respectively. The increase in colony number caused by NRG1 is impaired by ibrutinib. The increase in colony number caused by GRP was impaired by PD176252 or ibrutinib. The results indicate that GRP or NRG1 stimulate proliferation, whereas PD176252 or ibrutinib reduce proliferation of NSCLC cells.

### 3.10. HER4 Splice Variants

**Figure 10:**
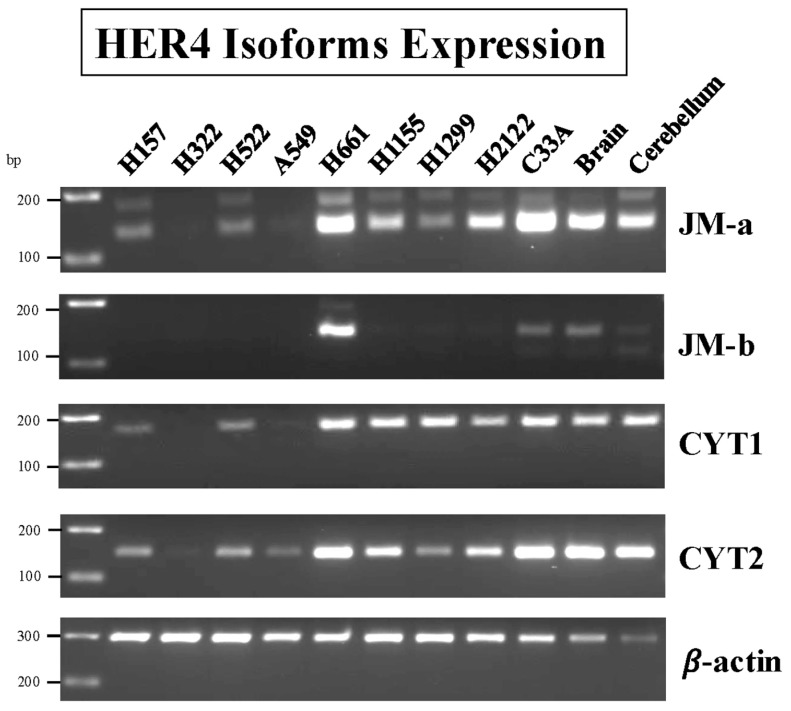
Expression of HER4 Isoforms in human NSCL cancer cell lines. RT-PCR was performed with 8 human lung cancer cell lines, one cervix cancer cell line (C33A), brain and cerebellum to evaluate the expression of HER4 Isoforms (JM-a, JM-b, CYT1, and CYT2) mRNA. β-Actin was used as a loading control. Primers used are shown in Table 1, and experimental conditions are as described in Materials and Methods. This experiment is representative of 2 others. Abbreviations: CYT, cytosolic C-terminus; JM, extracellular juxtamembrane region. JM-a, JM-b, CYT1, and CYT2 are HER4 splice variants. The original western blot images can be found in Supplementary Figure S9.

HER4 is unique among the ErbB family of tyrosine kinases in possessing four splice variant (SV) isoforms [3,4,19,69]. At position 624 of HER4, in the extracellular juxtamembrane region, a 26-amino-acid SV is added (JM-a) or a different 16-amino-acid peptide (JM-b). At position 1044 of HER4, in the cytosolic C-terminus, distal to the tyrosine kinase domain, a 20 amino acid peptide is added (CYT1), whereas there is no addition in CYT2. JM-a/CYT1 and JM-a/CYT2 are cleaved by TACE/ADAM17, but not JM-b/CYT1 and JM-b/CYT2 [4,69]. Figure 10 shows that the highest amount of JM-a mRNA is present in NCI-H661 and NCI-H2122 and the cervical cancer cell line, C33A cells, as well as the positive controls, brain and cerebellum.

Moderate concentrations of JM-a are present in NCI-H1155 and NCI-H2122 cells, minimal amounts in NCI-H157, H522, and H1299 cells, and none was found in NCI-H322 or A549 cells (Figure 10). High concentrations of JM-b are present only in NCI-H661 cells. High concentrations of CYT1 are present in NCI-H661, NCI-H1155, NCI-H1299, and NCI-H2122 cells. High concentrations of CYT2 are present in NCI-H661, NCI-H1155, and NCI-H2122 cells, whereas moderate concentrations were present in NCI-H157, NCI-H522, and NCI-H1299 cells. Cell line NCI-H661 has abundant mRNA for JM-a, JM-b, CYT1, and CYT2, whereas H522 cells had low amounts of JM-a, CYT1, and CYT2, but no JM-b. The results indicate that all HER4 SVs are present in different NSCLC cells.

In Table 4, the effects of MEK and PI3K/AKT inhibitors were investigated on NCI-H661 NSCLC cells.

Adding 10 μM PD98059 or LY294002 to NCI-H661 cells had little effect on basal P-HER4, P-ERK, or P-AKT. PD98059, but not LY294002, inhibited the growth of NCI-H661 cells. Adding 0.1 μM GRP to NCI-H661 cells increased P-HER4, P-ERK, and P-AKT significantly. The increase in P-ERK, but not P-AKT or P-HER4, caused by GRP addition was inhibited by PD98059. The increase in P-AKT, but not the increase in P-ERK or P-HER4, caused by GRP addition, was inhibited by LY194002. The results demonstrate that the effect on HER4 is upstream from MEK and PI3K and support the conclusion that activation of the MEK/ERK pathway, but not the PI3K/AKT pathway, is important for P-ERK activation, which is an important mediator of proliferation.

## 4. Discussion

Non-small cell lung cancer (NSCLC), which kills approximately 130,000 United States patients annually, is often treated with platinum chemotherapy; however, the 5-year survival rate is only 16% [70]. Immunotherapy with pembrolizumab has improved the therapy for some NSCLC patients [71]. A large number of studies show that activation of the ErbB family of receptors (particularly EGFR/HER1, HER2, and HER3) plays pivotal roles in the development, pathogenesis, and behavior of NSCLC cells [72]. In NSCLC cells the ErbB family plays these pivotal roles by ligand activation, due to the release of cognate ligands, often due to their transactivation by GPCRs, resulting in stimulation of a number of potent tumor growth cascades (i.e., MAPK, PI3K/AKT, STAT, mTor, RAS, and RAF cascades) [1,29,72]. Furthermore, in NSCLC cells constitutive activation of ErbBs occurs frequently due to overexpression of the ErbB family or the presence of mutant members of the ErbB family, causing ligand-independent signaling [2,23,73]. Patients with L858R EGFR mutations can be treated with TKIs, such as gefitinib or erlotinib [1,27].

The role of HER4, the newest member of the ErbB family, has been less well studied in NSCLC. Extensive studies involving EGFR/HER1, HER2, and HER3 in NSCLC cells demonstrate that each of these ErbB s, can be transactivated in a ligand-dependent manner through activation of G protein-coupled receptors (GPCRs) on NSCLC cells, especially by members of the Bombesin (Bn) receptor family (Gastrin-releasing peptide (GRPR/BB2R) and Neuromedin B receptors (NMBR/BB1)), which are present on >75% of lung cancer cell lines [33,37,74,75]. Because there is no information on whether a similar situation exists in NSCLC cells with HER4, the present study was performed.

In the present study we found that EGFR, HER2, HER3, and HER4 mRNA were present in most of the 12 NSCLC cell lines studied (Figure 1). This result is similar to a number of other studies with EGFR/HER1, HER2, and HER3 in NSCLC cells [20,37,52,53,74], but because these NSCLC cancer cell lines were preselected due to at least one study reporting they contained HER4, our result differs from most studies with HER4, which generally show HER4 protein present in 30–40% of NSCLC cell lines [20,41,48,49,50,51,52,53,54,55,76], even though in PCR studies HER4 mRNA is present in almost all NSCLC cells [57]. In our study, HER4 and one of its native ligands, neuregulin (NRG1), were also present in most NSCLC cell lines (Figure 2).

In the present study, we also found that the Bn receptor GRPR was present in most of the NSCLC cell lines studied, as reported previously by others [33,37,74,75,77]. The GRPR contains 384 amino acids with a 38 amino acid *N*-terminal, 7 transmembrane (TM) domains, and a 58 amino acid C-terminal [33]. Bn or GRP binds to the GRPR extracellular loops 2 and 3, as well as TM3, TM6, and TM7 at the top of a binding pocket [78,79]. The BnR peptoid antagonist, PD176252, binds to amino acids Ser179, Glu175, and Arg308 of the GRPR at the bottom of the binding pocket [80]. The GRPR intracellular loop 3 interacts with Gq, leading to increased Phosphoinositide (PI) metabolism [33]. Adding Bn to NSCLC cells increases P-HER4 in a time- and dose-dependent manner (Figure 3). The increase in cytosolic Ca^2+^ caused by GRP addition is blocked by the peptide receptor antagonist PD176252. PD176252 antagonizes the NMBR in addition to the GRPR [60,61], but BW2258U89 is specific for the GRPR [33]. In the present study, adding GRP to NCI-H661 cells increased transactivation of HER4, which was inhibited by the BnR antagonist, PD176252, or the GRPR-specific antagonist, BW2258U89 (Figure 4).

The GRPR causes transactivation of the EGFR [36]. Adding GRP to head and neck cancer cells HNSCC causes Src-dependent cleavage of EGFR ligands [81]. The growth of head and neck squamous cell carcinoma (HNSCC) is inhibited by erlotinib (TKI) or PD176252. Adding both gefitinib and PD176252 inhibited the growth of cancer cells better than either agent alone [30]. Adding GRP to NSCLC cells increases PY1068-EGFR, PY1248-HER2, and PY1289-HER3 [41]. Adding GRP to NSCLC cells increased P-HER4, which is inhibited by siRNA HER4 (Figure 5). The increase in PY-HER4 caused by GRP was inhibited by the GRPR receptor antagonist, PD176252, and the tyrosine kinase inhibitor, ibrutinib.

ROS species are essential for GPCR regulation of HER4 transactivation. Figure 6 shows that the ROS inhibitors *N*-Acetylcysteine (NAc is an antioxidant) and Tiron (Tir is a superoxide scavenger) inhibit the ability of the GRPR to stimulate transactivation of HER4 and the formation of HER4 homodimers or HER2/HER4 heterodimers. NADPH oxidase and Dual Oxidase (DUOX) are present in NSCLC cells, and the enzymes produce reactive oxygen species (ROS) [82]. The ROS can oxidize essential Cys amino acids in protein tyrosine phosphatases, reducing enzymatic activity [83]. ROS inhibitors decrease phosphorylation of receptor tyrosine kinases (RTK), such as the EGFR (Figure 6). Diphenylene iodonium is a NOX and DUOX inhibitor, which inhibits cancer proliferation and RTK transactivation [84].

Src is phosphorylated after cellular treatment with GRP [85]. Src can activate ADAM-17, which cleaves growth factors from inactive precursors to active growth factors, increasing RTK activity [31]. NRG1 and NRG3 mRNA are present in many NSCLC cell lines (Figure 8). NRG1 is stored in the cellular membrane as a 640 amino acid precursor protein, which is metabolized by ADAM-17. Cleavage with ADAM-17 results in the secretion of a 222 amino acid protein, which has an EGF-like domain that can activate HER3 or HER4 [86]. Table 2 shows that NRG1 secretion is increased by the agonist GRP but inhibited by the GRPR antagonist, PD176252. Figure 7 shows that GRP and NRG1 increase PY1284-HER4. Because NCI-H522 and H661 cells have low HER3, the NRG1 primarily activates HER4 in these cells. A unique property of NRG1 is that it forms fusion partners with other proteins such as CD74 and ATP1B1 [87]. Because CD74-NRG1 has an extracellular EGF-like domain, it promotes the formation of HER2-HER4 heterodimers [88,89]. When patients have NRG1 fusion proteins, they have an increased response to afatinib (a tyrosine kinase inhibitor, TKI) and other chemotherapeutic agents [6,20]. Approximately 10% of the invasive mucinous adenocarcinoma patients have NRG1 fusions.

HER4 mutations have been identified in approximately 5% of the NSCLC patients. Activating HER4 mutations occur at Y285C and D595V, which are in the extracellular ligand binding domain, as well as D931Y and K935I, which are in the kinase domain. The extracellular domain HER mutations facilitate formation of HER4 homodimers as well as HER2/HER4 heterodimers [21]. The Y285C, D595V, and K935I mutations promote survival of serum-starved NSCLC cells. HER4 mutants have increased proteolysis, resulting in elevated HER4 intracellular domain (ICD). Figure 10 shows that cell line NCI-H661 has high levels of the JM-a and CYT1 mRNA. JM-a CYT1 is metabolized to 4ICD, which can lead to cellular proliferation.

Ibrutinib, a TKI with high affinity for Bruton tyrosine kinase, is used in the treatment of chronic lymphocyte leukemia and has been shown to also inhibit HER4 and other TKs [6,17]. Ibrutinib inhibits the growth of NSCLC cell lines with high affinity if the cells have low Wnt5 (NCI-H522 and NCI-H661), but not cells with high Wnt5 (NCI-H146 and A673 [65]). Ibrutinib increases the ability of PD16252 to inhibit NSCLC proliferation. Figure 9 shows that Ibutinib inhibited the growth of NCI-H522 and NCI-H661 with high affinity. Furthermore, GRP and NRG1 stimulate colony formation of NSCLC cells (Table 3), whereas PD176252 or ibrutinib inhibits proliferation. The results indicate that the activation of GRPR results in HER4 transactivation, which can stimulate NSCLC proliferation.

Strong roles have been established for activation of EGFR/HER1, HER2, and HER3 in NSCLC cell growth, aggressiveness, survival, and as prognostic factors. The role of HER4 has remained unclear with conflicting data. One of the unique characteristics of HER4, in contrast to the other three ErbB members, is that in some tumors HER4’s activation results in inhibition of tumor growth/tumor proliferation/aggressiveness (breast, pancreatic, bladder, and colon) [6,17,90,91], and its presence can be predictive of improved survival and/or favorable clinical-pathological features [3,18,92,93]. Our results support the conclusion that HER4 activation, whether stimulated by GRPR activation or NRG1, can stimulate the growth of NSCLC cells (Table 3). This result is consistent with a number of other studies in NSCLC cancer cells, which concluded that activation of HER4 can stimulate their growth [20,21,49,52,94,95]. These results differ from studies that report that higher expression levels of HER4 did not correlate with clinical indicators of lung tumor growth, such as tumor recurrence, development of distal metastases, or survival [54,96,97]. Our results provide for the first time insights into the signaling cascades mediating the HER4-stimulated growth of NSCLC cells. Similar to findings with other members of the ErbB family (EGFR/HER1, HER2, HER3), our study shows HER4 activation in NSCLC cancer cells can result in growth cell mediated by activation through GPCR transactivation or by direct ligand activation by ErbB member ligands through a ROS-mediated process. Furthermore, our studies show that HER4 activation can stimulate both the MAPK and the activation of PI3K/AKT, which are well-established growth-mediating cascades in other tumors [98,99]. However, in our study only the activation of the MAPK cascade mediates NSCLC cell proliferation (Table 4). This result is somewhat surprising in light of a number of other findings in NSCLC and other cancers. First, HER4 has been shown in other tumor cells to activate PI3K and the MAPK cascade [6,12,69,100,101,102] and, in osteosarcoma cells [100], neuroblastomas [103], gliomas [16], or gastric cancer cells [12], to promote growth and/or development of metastases by a HER4-mediated PI3K activation mechanism. Second, activation of the PI3K/AKT is reported in 51–74% of NSCLCs; it is one of the principal growth-stimulating signals in these cells, and its activation correlates with invasiveness and progressive growth [104]. Third, a number of studies, in both lung cancer cells and other cells, report that activation of EGFR1/HER1, HER2, and HER3 results in stimulation of the MAPK cascade, as well as activation of the PI3K/AKT pathway, each of which can have an important effect on cell behavior (growth, migration, apoptosis, and biological response). In many cases, such as in breast cancer, the activation of the PI3K cascade is particularly important for growth, tumor migration, and TKI growth resistance [98,99,105,106,107]. Our results differ from the results reported in many of these studies involving both tumor and nontumor cells reporting that PI3K activation, not MAPK activation, is the principal downstream effector of HER2/HER3-signaling on cellular growth [106,108,109,110], with EGFR homodimers as well as EGFR/HER2 heterodimer complexes preferentially activating MAPK signaling and HER2/HER3 heterodimers to a lesser extent [106,111,112]. Similar to our results, MAPK inhibitors were more effective than PI3K inhibitors at inhibiting HER3-mediated growth in several studies with HNSCC [106,112] and breast cancer [113,114,115,116]. Our results are also similar to studies in NSCLC cells, which reported activation of bombesin receptors [41] resulted in transactivation of the HER3, causing activation of both the MAPK and PI3K/AKT signaling cascades; however, only activation of the MAPK-dependent mechanism was important in mediating cell growth. Similarly, our results are similar to those in gastric cancer reporting that gastric cancer migration due to HER3/HER4 transactivation, caused by histone deacetylase inhibitors, was mediated by MAPK activation, but not PI3K/Akt activation [117]. Lastly, our results are compatible with recent studies, which report in patients with NSCLC the encouraging therapeutic results shown by treatment strategies involving the combination of MEK inhibitors with other therapies such as BRAF inhibition, immunotherapy or chemotherapy [118]. Our result with GRPR/NRG1 activation of HER4, mediating NSCLC cell growth via a MAPK-mediated mechanism, differs from studies that report that thrombopoietin [119], muscarinic M3 cholinergic receptor activation, and GRPR activation [120] stimulated transactivation of EGFR/HER1 in NSCLC cells, which mediated growth, invasion, or migration through a PI3K/AKT-dependent mechanism [121]. They also differ from studies that report that growth and resistance to gefitinib in NSCLC cells are mediated by signaling through the EGFR/HER1 activation by a PI3K-dependent mechanism [122]. These results demonstrate that the signaling cascades, mediating the growth effects of different stimuli through activation of the different ErbB members, can vary markedly in the same and different tumor cells.

## 5. Conclusions

The GRPR regulates transactivation of HER4 in a ligand- and time-dependent manner. The increase in P-HER4 caused by GRP is impaired by both the GRPR antagonist, PD176252, and the TK inhibitor, ibrutinib. Adding GRP to non-small cell lung cancer (NSCLC) cells causes the secretion of NRG1, which activates HER4. Activated HER4 can form HER4 homodimers or HER4-HER2 heterodimers in a ROS-dependent manner. The growth of NSCLC cells is stimulated by GRP or NRG1 but inhibited by PD176252 or ibrutinib in vitro. It remains to be determined if a combination of PD176252 and ibrutinib will inhibit the growth of NSCLC in vivo.

## Figures and Tables

**Table 1:** PCR primers and PCR assay conditions.

Gen	Primer	Sequence	Product Size
EGFR	Forward	5′-TCTTCGGGGAGCAGCGAT-3′	119
	Reverse	5′-TCGTCGCCTTGGCAAACTTTC-3′	
HER2	Forward	5′-AGCCGCAGTGAGCACCATGG-3′	102
	Reverse	5′-GTGCCGGTGCACACTTGGGT-3′	
HER3	Forward	5′-CCTATGCAGGGCTACGATTGG-3′	131
	Reverse	5-GTTGGGCTCAGCAGGTAACT-3′	
HER4	Forward	5′CATTTGACCATGACCATGTAAACGTC-3′	135
	Reverse	5′-GGAACTGATGACCTTTGGAGGAA-3′	
NRG1	Forward	5′-TCGGTGTGAAACCAGTTCTGA-3′	130
	Reverse	5′-GCGAAGTTCTGACTTCCCTG-3′	
NRG2	Forward	5′-GCTGGTACCCCACTGATGATGAC-3′	232
	Reverse	5′-CACATCACCCCAGAGTGGAG-3′	
NRG3	Forward	5′-AGTCAAGTTTTGTCGGCCCC-3′	93
	Reverse	5′-AAGAGGATAGACTCCTGTGGTG-3′	
NRG4	Forward	5′-ATCAGTCCATCGGCAACTGCTA-3′	116
	Reverse	5′-ACATTTGCCTCTGGTTGCTTC-3′	
JM-a	Forward	5′-GAAATGTCCAGATGGCCTACAGGG-3′	175
	Reverse	5′-AATGCAGTCATGACTAGTGGGACC-3′	
JM-b	Forward	5′-CCACCCATCCCATCCAAA-3′	180
	Reverse	5′-CCAATTACTCCAGCTGCAATC-3′	
CYT1	Forward	5′-GGATGAAGAGGATTTGGAAG-3′	145
	Reverse	5′-TCCTGACATGGGGGTGTA-3′	
CYT2	Forward	5′-GAATAGGAACCAGTTTGTATACCG-3′	205
	Reverse	5′-ACAGCAGGAGTCATCAAAAATC-3′	
β-actin	Forward	5′-CCTCGCCTTTGCCGATCC-3′	205
	Reverse	5′-GGAATCCTTCTGACCCATGC-3′	

Amplification for all PCR reactions included an initial cycle of 95 °C for 15 min, followed by 35 cycles of denaturation at 94 °C for 30 s, annealing at 60 °C for 30 s, and extension at 72 °C for 1 min. After the final cycle, all PCR reactions concluded with a 10 min extension at 72 °C. Abbreviations: CYT, cytosolic C-terminus; JM, extracellular juxtamembrane region; NRG, Neuregulin; JM-a, JM-b, CYT1, and CYT2 are HER4 splice variants.

**Table 2:** NRG1 secretion from NSCLC cells.

	NCI-H522 Cells		NCI-H661 Cells	
Addition	NRG1β (pg/mL)	*p*-Value	NRG1β (pg/mL)	*p*-Value
None	140 ± 16		152 ± 21	
GRP, 0.1 μM	235 ± 29	*p* = 0.02 vs. None	322 ± 41	*p* = 0.004 vs. None
GRP + PD176252, 1 μM	162 ± 22	*p* = 0.05 vs. GRP, 0.1 μM	217 ± 27	*p* = 0.03 vs. GRP, 0.1 μM

PD176252 (1 μM) was added to NCI-H522 and NCI-H661 cells for 30 min, followed by the addition of 0.1 μM GRP for 10 min. Results are expressed as pg/mL of NRG1β under each condition. The mean value ± S.E. of 3 determinations is shown, each repeated in duplicate. Abbreviations: GRP, Gastrin-Releasing Peptide; NRG, Neuregulin; NSCLC, non-small cell lung cancer.

**Table 3:** Effects of MEK and PI3K inhibitors in the clonogenic growth assay of NCI-H661 cells.

	Colony Number		+0.1 μM GRP	
Addition	Mean ± S.D.	*p*-Value	Mean ± S.D.	*p*-Value
None	27 ± 3		54 ± 6	*p* = 0.006 vs. None
PD176252, 10 μM	16 ± 3	*p* = 0.02 vs. None	33 ± 5	*p* = 0.009 vs. + 0.1 μM GRP
Ibrutinib, 10 μM	21 ± 4	*p* = 0.06 vs. None	41 ± 7	*p* = 0.07 vs. + 0.1 μM GRP
PD + Ibrutinib	12 ± 5	*p* = 0.02 vs. None	26 ± 4	*p* = 0.005 vs. + 0.1 μM GRP
siRNA HER4	9 ± 3	*p* = 0.001 vs. None	19 ± 5	*p* = 0.001 vs. + 0.1 μM GRP
NRG1, 10 ng/mL	60 ± 7	*p* = 0.004 vs. None	n.d.	
NRG1 + Ibrutinib	39 ± 6	*p* = 0.05 vs. None *p* = 0.02 vs. NRG1	n.d.	

Results are expressed as the colony number of NCI-H661 cells determined as described in Materials and Methods. The mean value ± S.D. of 3 determinations using NCI-H661 cells. Abbreviations: GRP, Gastrin-Releasing Peptide; n.d., not determined; NRG, Neuregulin; PD, PD176252.

**Table 4:** Effects of MEK and PI3K inhibitors in the MTT growth assay of NCI-H661 cells.

	P-HER4		P-ERK		P-AKT		Proliferation	
Addition	Mean ± S.D.	*p*-Value	Mean ± S.D.	*p*-Value	Mean ± S.D.	*p*-Value	Mean ± S.D.	*p*-Value
None	100 ± 6		100 ± 7		100 ± 8		100 ± 5	
GRP, 0.1 μM	284 ± 15	*, *p* = 0.001	216 ± 13	*, *p* = 0.001	148 ± 12	*, *p* = 0.02	n.d.	
PD, 10 μM	95 ± 6	#, *p* = 0.001	92 ± 7	#, *p* = 0.001	103 ± 9	#, *p* = 0.05	72 ± 6	*, *p* = 0.013
LY, 10 μM	103 ± 9	#, *p* = 0.007	105 ± 6	#, *p* = 0.003	92 ± 8	#, *p* = 0.02	104 ± 7	
GRP + PD	246 ± 13	*, *p* = 0.001 #, *p* = 0.07	113 ± 6	#, *p* = 0.007	152 ± 14	*, *p* = 0.01 #, *p* = 0.76	n.d.	
GRP + LY	293 ± 16	*, *p* = 0.001	224 ± 18	*, *p* = 0.002	104 ± 5	#, *p* = 0.04	n.d.	

The ability of 0.1 μM GRP to stimulate P-HER4, P-ERK, and P-AKT in NCI-H661 cells was determined by Western blot. The ability of 10 μM PD 98,059 and LY294002 to impair proliferation of NCI-H661 cells was determined using the MTT assay. The mean value ± S.D. is indicated for 3 determinations. *p*-values; * vs. None and # vs. GRP. Abbreviations: GRP, Gastrin-Releasing Peptide; n.d., not determined; P-AKT, PS473AKT; P-ERK, PT202/Y204-ERK; P-HER4, PY1284-HER4; PD, PD98059. LY, LY294002.

## Data Availability

The raw data supporting the conclusion of this article will be made available by the authors without undue reservation.

## Supplementary Materials

The following supporting information can be downloaded at: https://www.mdpi.com/article/10.3390/biology14091225/s1.

## Abbreviations

The following abbreviations are used in this manuscript:

Bn, bombesin; BW, BW2285U89; CYT, cytosolic C-terminus; ECD, extracellular domain; GPCR, G-protein-coupled receptor; GRP, gastrin-releasing peptide; GRPR/BB2R, Gastrin-releasing peptide receptor; I, ibrutinib; ICD, intracellular domain; JM, juxtamembrane region; NAc, *N*-acetylcysteine; NMB, neuromedin B; NMBR/BB1R, Neuromedin B receptor; NRG, neuregulin; NRGs, neuregulin’s, NSCLC, non-small cell lung-cancer cells; P, phosphorylation; PD, PD176252; PI, phosphatidylinositol; PK, protein kinase; ROS, reactive-oxygen species; RTK, receptor-tyrosine kinase; STAT, signal transducer and activator of transcription; SV, splice variant; SVs, splice variants; Tir, Tiron; TKI, tyrosine kinase inhibitor; TM, transmembrane.
