# Supplementary material for: Gastrin-Releasing Peptide Receptors Stimulate MAPK-Mediated Growth of Lung Cancer Cells by Transactivating HER4 in a Neuregulin-1, MAP Kinase-Dependent Manner Requiring Activation of the ROS-System
Source: Biology (Basel). 2025 Sep 9;14(9):1225. doi: 10.3390/biology14091225 (PMC12467189; doi:10.3390/biology14091225)

Supplementary Figures. Originals blots shown in this study.

Figure S1. Original blots.

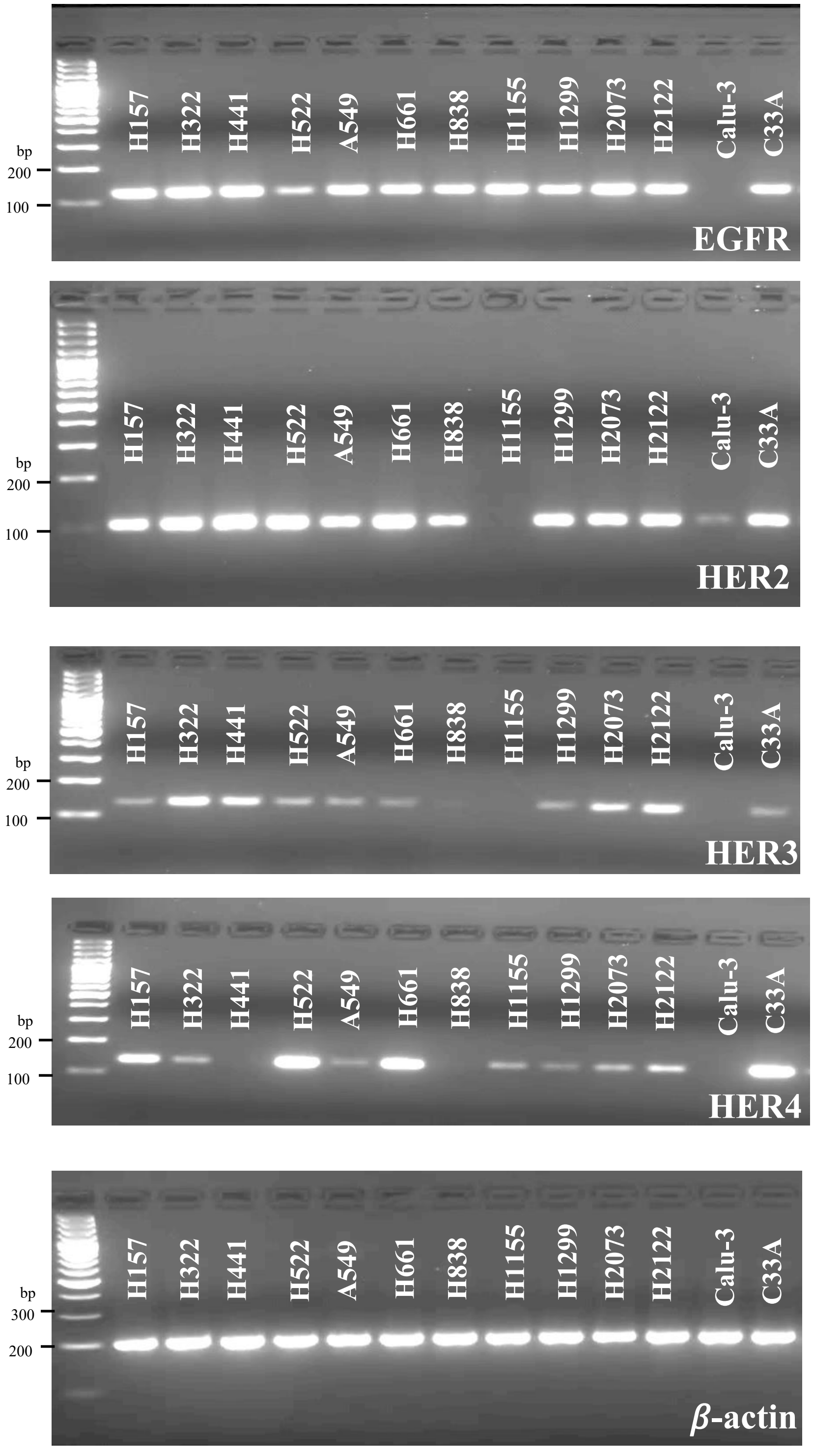

Figure S2. Original blots.

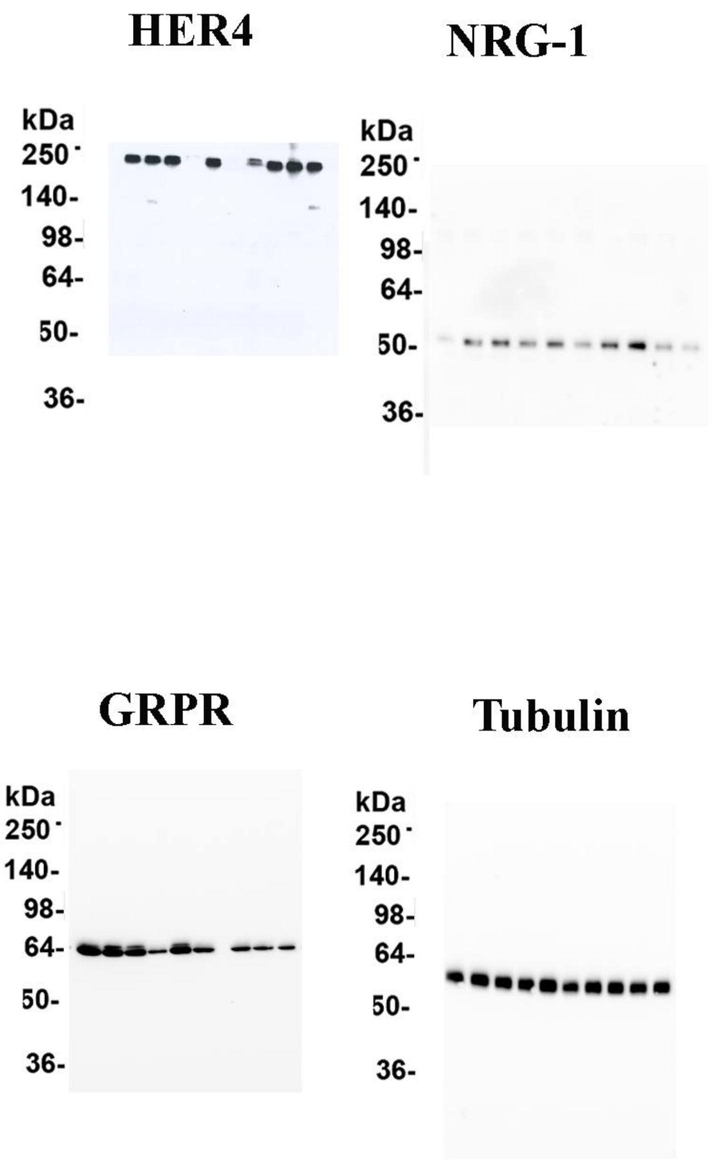

Figure S3. Original blots.

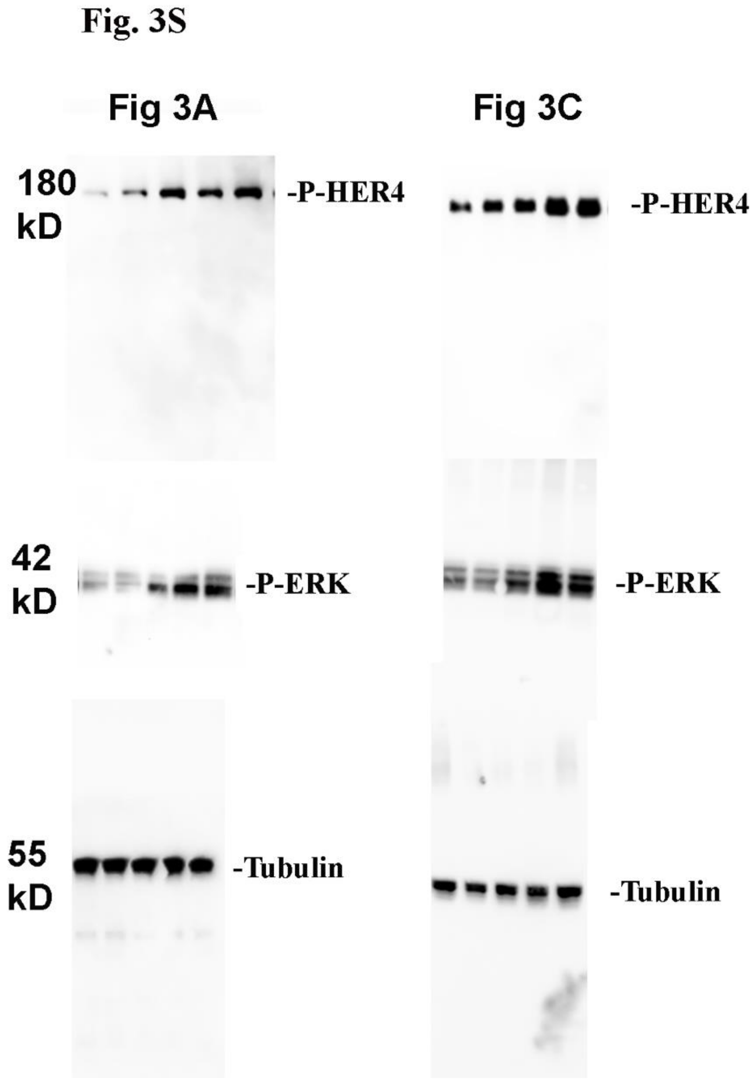

Figure S4. Original blots.

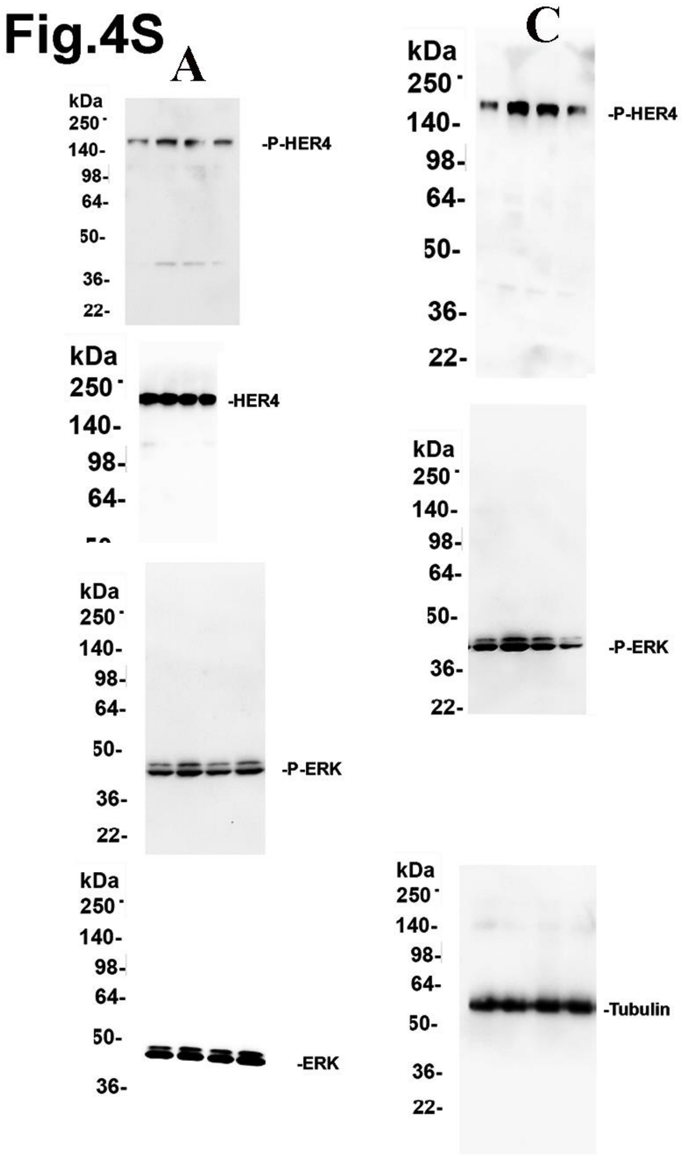

Figure S5. Original blots.

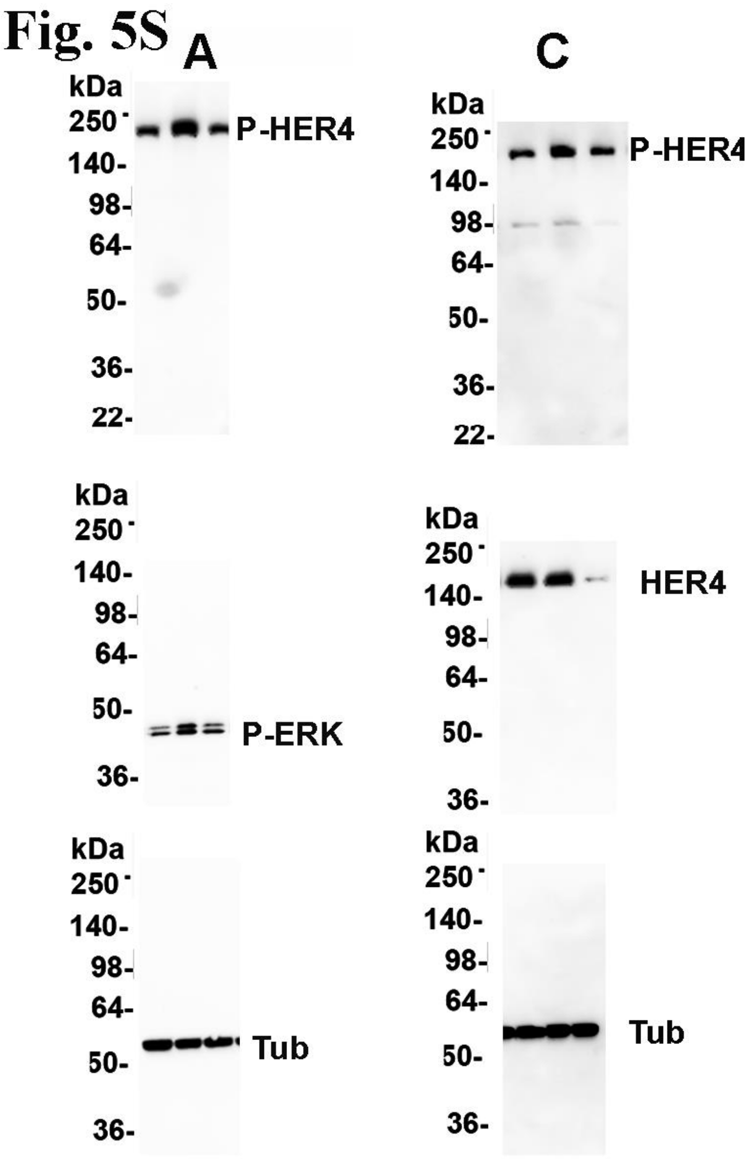

Figure S6. Original blots.

Fig. 6s

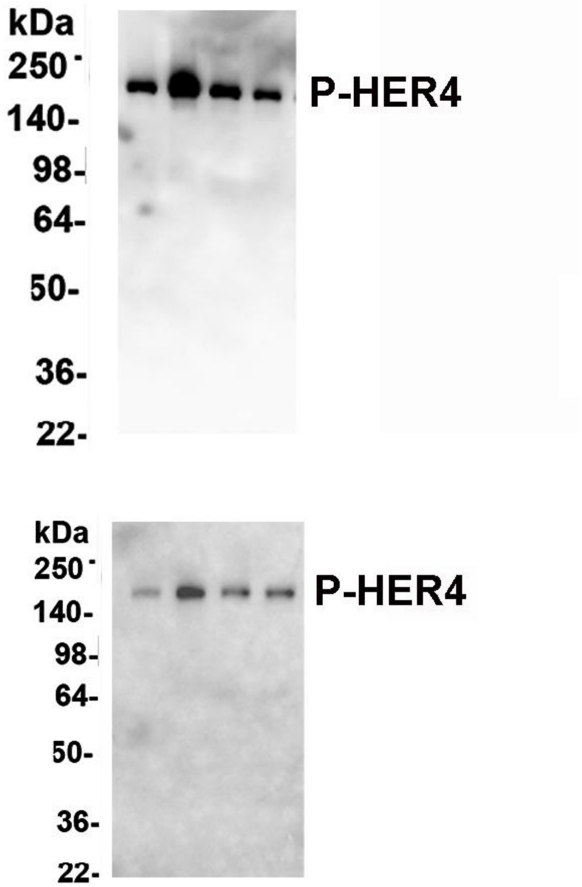

Figure S7. Original blots.

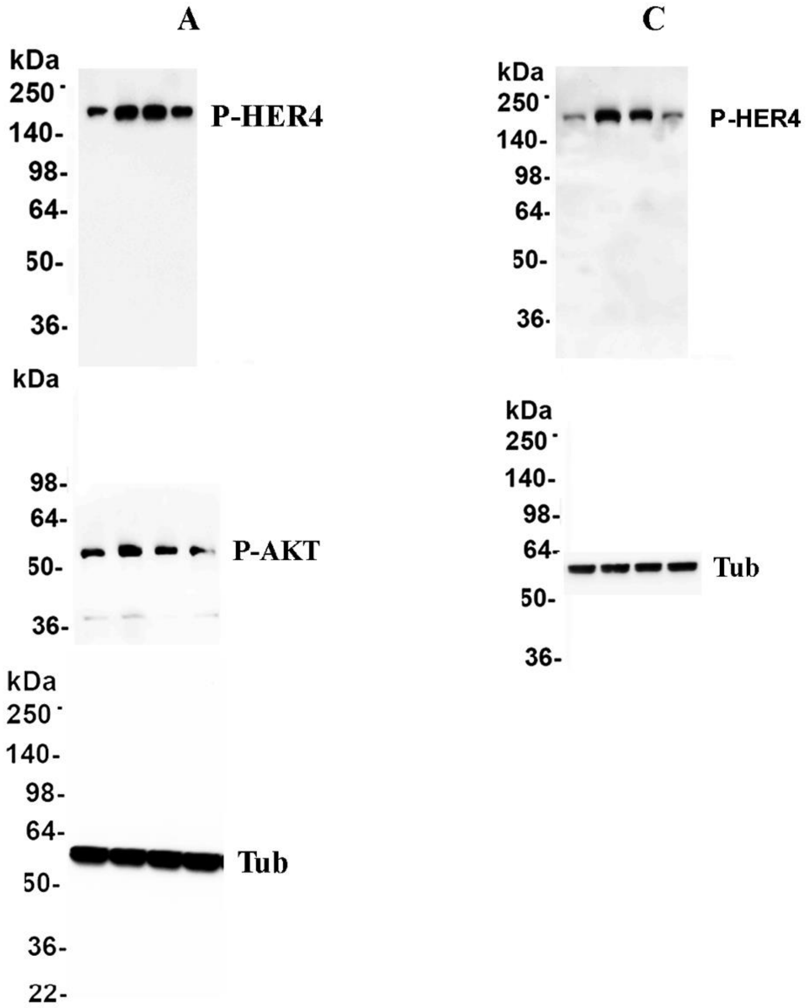

Figure S8. Original blots.

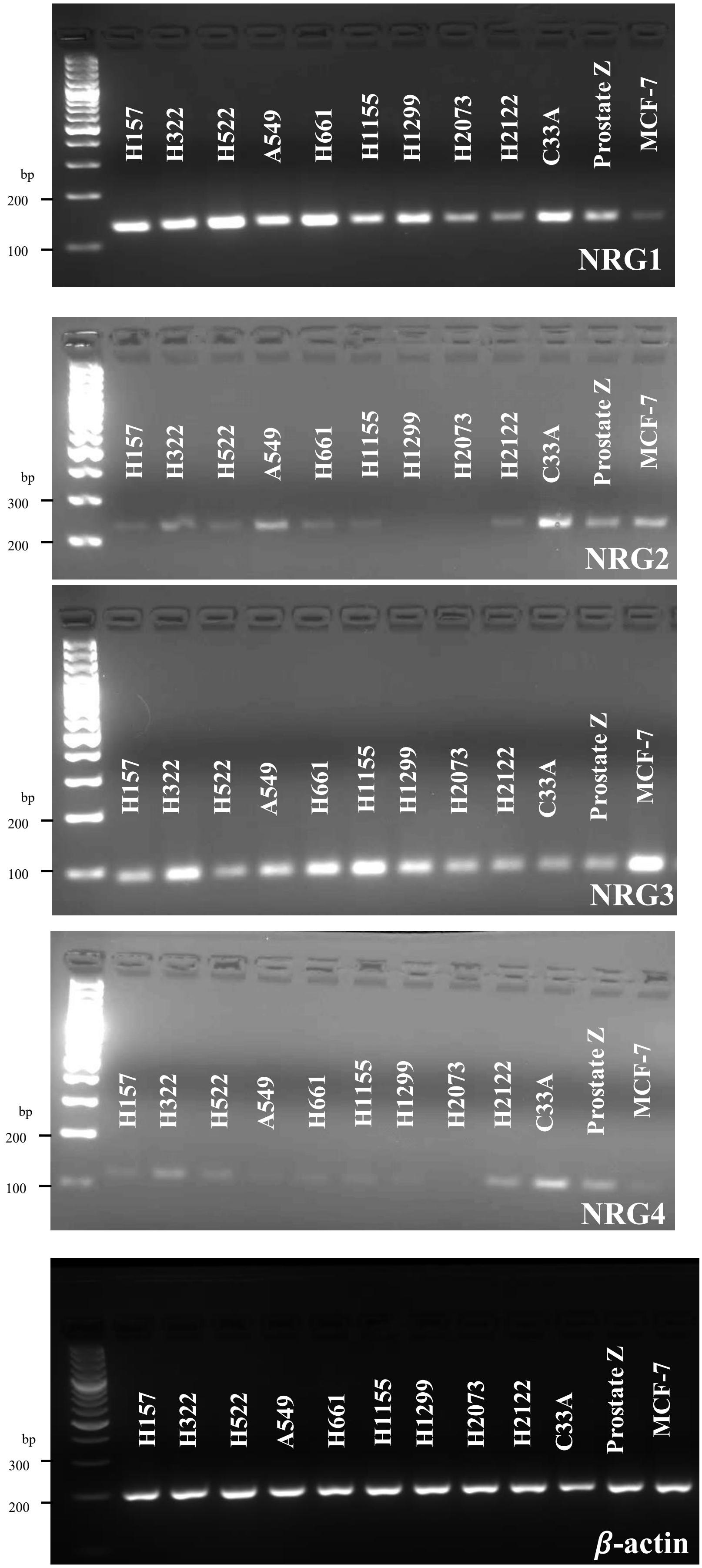

Figure S9. Original blots.

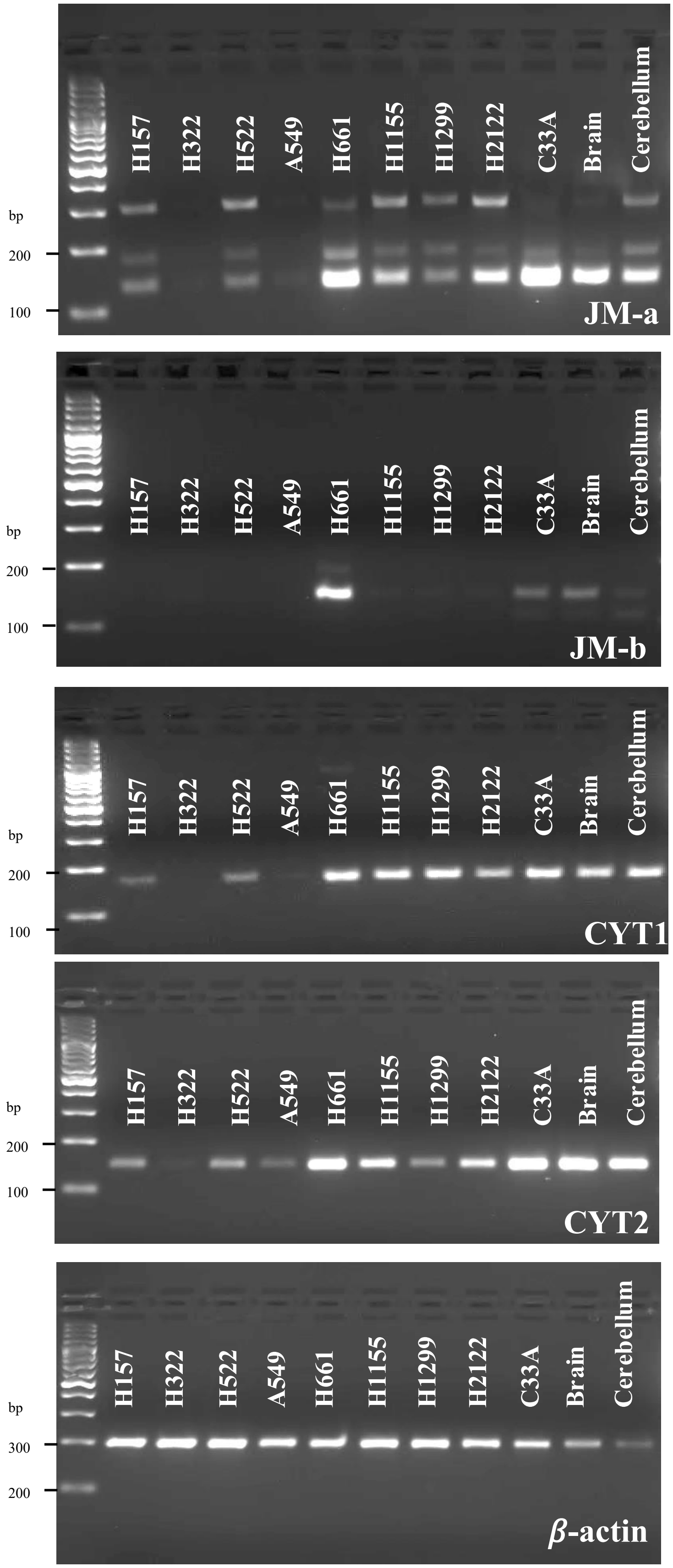

Supplement: Supplementary file 1 [file biology-14-01225-s001.zip › biology-3789968-supplementary.pdf]
